# Atmospheric circulation over Europe during the Younger Dryas

**DOI:** 10.1126/sciadv.aba4844

**Published:** 2020-12-11

**Authors:** Brice R. Rea, Ramón Pellitero, Matteo Spagnolo, Philip Hughes, Susan Ivy-Ochs, Hans Renssen, Adriano Ribolini, Jostein Bakke, Sven Lukas, Roger J. Braithwaite

**Affiliations:** 1 School of Geosciences University of Aberdeen, Aberdeen, UK.; 2 Departamento de Geografía, Universidad Nacional de Educación a Distancia (UNED), Madrid, Spain.; 3 Department of Geography, University of Manchester, Manchester, UK.; 4 Laboratory of Ion Beam Physics, ETH Zürich, 8093 Zürich, Switzerland.; 5 Department of Natural Sciences and Environmental Health, University of South-Eastern Norway, Bø, Norway.; 6 Dipartimento di Scienze della Terra, Università di Pisa, Via S. Maria 53, 56126, Pisa, Italy.; 7 Department of Earth Science, University of Bergen, P.O. Box 7800 5020 Bergen, Norway.; 8 Department of Geology, Lund University, Sölvegatan 12, Lund, Sweden.

## Abstract

The Younger Dryas (YD) was a period of rapid climate cooling that occurred at the end of the last glaciation. Here, we present the first palaeoglacier-derived reconstruction of YD precipitation across Europe, determined from 122 reconstructed glaciers and proxy atmospheric temperatures. Positive precipitation anomalies (YD versus modern) are found along much of the western seaboard of Europe and across the Mediterranean. Negative precipitation anomalies occur over the Fennoscandian ice sheet, the North European Plain, and as far south as the Alps. This is consistent with a more southerly and zonal storm track, which is linked to a concomitant southern location of the Polar Frontal Jet Stream, generating cold air outbreaks and enhanced cyclogenesis, especially over the eastern Mediterranean. This atmospheric configuration resembles the modern Scandinavian (SCAND) circulation over Europe (a blocking high pressure over Scandinavia pushing storm tracks south and east), and by analogy, a seasonally varying palaeoprecipitation pattern is interpreted.

## Teaser

Reconstructed palaeoprecipitation reveals a persistent SCAND-like circulation and southerly storm track during the Younger Dryas.

## INTRODUCTION

The Younger Dryas (YD) is recognized as a period [12.9 to 11.7 thousand years ago (ka)] of rapid climate change (*1*–*4*), with the strongest impacts apparent in the Northern Hemisphere. Changes in the Atlantic Meridional Overturning Circulation are believed to be a major contributor (*4*), which could happen in the present day due to increased meltwater runoff and iceberg discharge from the Greenland Ice Sheet (*5*). An atmospheric circulation reorganization occurred during the YD, as indicated by proxy-derived air temperatures (*6*, *7*), but its nature has only been observed in numerical models (*4*, *7*, *8*). Compared to the Bølling-Allerød, the temperature recorded over the Greenland Ice Sheet cooled over periods of years to decades, and by up to 10°C (*9*), North Atlantic sea surface temperatures (SSTs) cooled by 1° to 7°C (*10*), and European climate was colder and/or drier with enhanced seasonality (*6*, *7*, *11*). Changes in the tropics were minor while Southern Hemisphere mid- to high-latitudes experienced warming (*4*). The opposite southern hemispheric climate trend indicates a regional, Northern Hemisphere, forcing of the YD. The rapidity of the cooling suggests an instantaneous (on the time scales and temporal resolution of proxy data) trigger with concomitant oceanic and atmospheric circulation reorganizations (*4*). In the Northern Hemisphere, the elevation of the ice sheets, and, particularly, the Laurentide ice sheet, appears to have controlled the large-scale hemispheric circulation pattern [the Polar Frontal Jet Stream (PFJS)] during the YD while ocean surface forcing (SST and sea ice) and the Fennoscandian ice sheet (FIS) mainly affected regional climate (*7*, *12*–*14*). However, the details of how climate, atmospheric circulation, and particularly precipitation changed during the YD remain elusive.

Climate proxies, generated from marine and terrestrial environments (*9*–*12*, *15*), may be used in combination with outputs from numerical climate simulations to investigate past climate dynamics (*8*, *15*). Marine sediments provide insight on large-scale processes such as meltwater pulses, but their temporal resolution is generally centennial at best. Terrestrial climate proxies tend to have higher temporal resolution (even sub-annual) but are often more indicative of local or regional conditions. Both marine and terrestrial proxies generally provide information on palaeotemperatures, which are useful for assessing the quality of numerical model outputs but are of limited use for identifying atmospheric circulation patterns beyond the summer months (*6*, *7*). Wind fields, derived from the orientations of relict sand dunes, have been used to assess atmospheric circulation patterns (*15*), but their geographical distribution is limited. Most terrestrial climate proxies can, at best, only be resolved in terms of wetter versus drier conditions, which is a function of precipitation, evaporation (temperature), and evapotranspiration (vegetation and temperature). Quantitative palaeoprecipitation estimates derived using palaeoglacier equilibrium line altitudes (ELAs) are a powerful tool for the assessment of regional-scale atmospheric circulation patterns, as precipitation is controlled by air mass advection at synoptic to sub-synoptic scales, and glaciers are, for the most part, little affected by sublimation and even less so by evaporation.

**Fig. 1:**
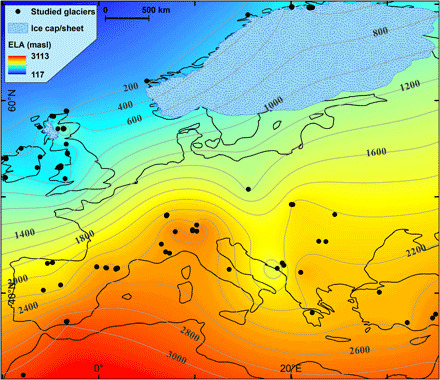
ELA_thl_ elevation surface for the YD. The ELA surface should be viewed as “theoretical” (ELA_thl_) because glaciers can only form where the topography is higher than the ELA_thl_ surface. For example, there are no glaciers in SE England or the low countries. Black dots show the location of palaeoglacier reconstruction sites. The FIS and the West Highlands icefield are shown, but the ice mass in the Alps is not shown due to incomplete knowledge of its geometry at this time.

In this study, reconstructed palaeoglaciers are used to derive a quantitative regional perspective on palaeoprecipitation, elucidating the atmospheric circulation over Europe during the YD. The ELA represents the elevation on a glacier surface where, at the end of the mass balance year (September in the Northern Hemisphere), annual accumulation (snowfall) equals annual ablation (snow and ice melt). The ELA is linked to atmospheric conditions via the interplay between temperature (ablation) and precipitation (accumulation) (*16*). Here, we present a unique dataset of ELAs and concomitant annual palaeoprecipitation at the ELA, calculated for 122 YD palaeoglaciers across Europe, from Morocco in the south to Norway in the north, and from Ireland in the west to Turkey in the east (Fig. 1). The results directly address ongoing debates on past hemispheric circulation and regional climate implications (especially precipitation) under different climate scenarios and provide an ideal palaeoclimate record to assess numerical model outputs (*17*).

**Fig. 2:**
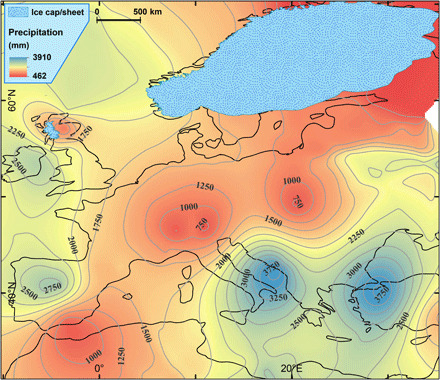
Potential total annual palaeoprecipitation at the glacier ELA during the YD (PPP_ELA_). The PPP_ELA_ was calculated using the proxy-derived SLE mean summer temperature (fig. S1) and converted to temperature at the ELA using a free-air lapse rate of 0.0065°C m^−1^. The ELA mean summer temperature was then used to derive the PPP_ELA_ (see Materials and Methods).

**Fig. 3:**
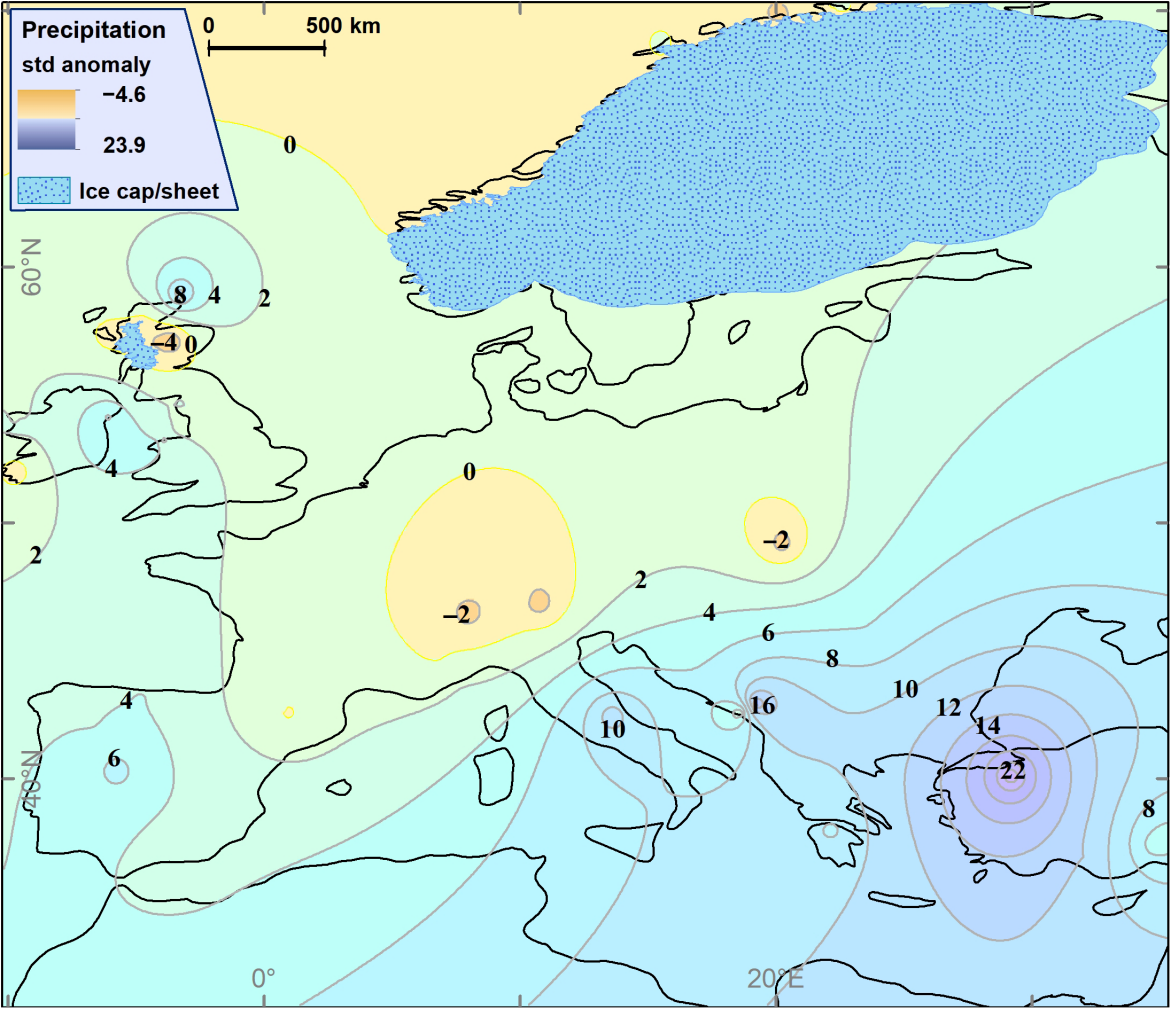
YD palaeoprecipitation anomalies at the ELA (PPA_ELA_). The precipitation anomalies identify regions of enhanced and reduced precipitation in comparison to the present day and most closely matches the m-SCAND circulation pattern. By analogy, the p-SCAND circulation would generate more precipitation in the spring and autumn along the northwestern seaboard as far north as the United Kingdom and in the western Mediterranean. The precipitation shifts south in mid-winter, in a zonal band across Iberia and the Mediterranean. The precipitation patterns are assumed to reflect the trajectory of the PFJS, which guides synoptic-scale depressions. By promoting cold air outbreaks over warmer oceans and seas, the PFJS provides conditions favorable for the formation of intense sub-synoptic low-pressure systems especially over the warm eastern Mediterranean. Note, however, that the positive anomalies in the Dinaric Alps and over Turkey are unrealistically high, which is a function of weaknesses in the modern gridded precipitation dataset (*39*) and not in the reconstructed YD palaeoprecipitation.

This study has applied rigorous chronological control to site selection (see Materials and Methods), unlike previous attempts to use palaeoglacier ELAs to study atmospheric circulation (*18*). An extensive search that identified moraines that had previously been dated to, or near to, the YD were initially investigated. Published dates were taken from the original papers except, where necessary, they were recalculated/recalibrated for terrestrial cosmogenic ^10^Be exposure ages and for ^14^C-dated organic samples (see Materials and Methods). Only moraines with an age that fell within the time span of the YD were selected for further analyses. The palaeoglaciers were then reconstructed from the dated frontal moraine, following an equilibrium profile flowline approach. This was extrapolated to a three-dimensional (3D) ice surface, from which ELAs were calculated, all using bespoke, semiautomated toolboxes in ArcGIS (see Materials and Methods). These data generated a regional, theoretical, ELA surface (ELA_thl_) (Fig. 1). Cosmogenic exposure ages have an uncertainty range such that the regional palaeoglacier reconstruction represents a YD “glacial maximum” snapshot. The YD has a colder first half and a warmer, more variable second half (*12*), so the assumption is made that the YD glacial maximum represents the first half of the YD (see Materials and Methods). A spatially comparable (overlapping the distribution of palaeoglaciers) mean summer temperature (June to August) dataset was developed using a range of marine and terrestrial proxies and converted to a sea level equivalent (SLE) temperature using a free-air lapse rate of 0.0065°C m^−1^. To maximize the number of data points and the spatial coverage of the temperature reconstruction, a single mean, for the first 500 years of the YD, for each site was determined (see Materials and Methods and fig. S1). Mean summer temperature at the palaeoglacier ELAs was determined using the same free-air lapse rate and used to calculate the annual “potential palaeoprecipitation” at the ELA (PPP_ELA_) (Fig. 2) (*16*), assuming that the glacier was in equilibrium with climate (see Materials and Methods). Palaeoprecipitation anomalies at the ELA (PPA_ELA_) have been calculated, compared to the modern precipitation (see Materials and Methods), to reveal the regional picture of enhanced and reduced precipitation across Europe during the YD (Fig. 3).

## RESULTS

### Equilibrium line altitudes

**Fig. 4:**
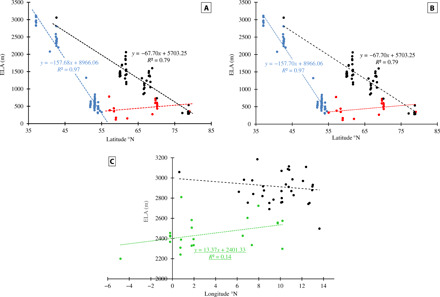
ELA gradients for the present day and the YD. The present-day ELAs are shown in black, and the piecewise regression fitted to the latitudinal transect of YD ELAs are shown in blue and red, south and north of the breakpoint, respectively. The longitudinal transect of YD ELAs across northern Spain and the Alps is shown in green. Regression equations are shown only for relationships, which are linearly related at the 90% (underlined) or 95% CIs. Present-day ELA data were obtained from the World Glacier Monitoring Service. (**A**) The piecewise regression was implemented in R, following an iterative searching approach, and located the breakpoint at 55.02°N. (**B**) The piecewise regression was implemented in R, using the segmented package, and located the breakpoint at 54.66°N. The decreasing to the north ELA gradients, both modern and YD, are assumed to represent temperature control on glacier mass balance and indicate a steeper gradient during the YD. The breakpoint in YD ELAs, located somewhere around 54.66°N to 55.02°N, should be taken as a transition zone, interpreted to be controlled by a reduction in precipitation north of the PFJS, and is taken to represent the average latitude of the PFJS during the first half of YD, over the western seaboard of Europe. (**C**) Comparison of the modern and YD ELAs in a west-east transect across the mountains of northern Spain and the Alps in a latitudinal band between 42°N and 47°N.

The ELA_thl_ surface (Fig. 1) shows a general northward decline, with complexity over the British Isles, and then a subtle rise in ELA northward, along western Norway, with the reversal centered on 55°N (see Materials and Methods). Plotting the ELAs from western Europe against latitude reveals a YD ELA gradient of approximately −157 m per degree of latitude (south of 55°N) and 9 m per degree of latitude (north of 55°N) compared with a present-day ELA gradient (from Spain to Svalbard) of approximately −68 m per degree of latitude (Fig. 4, A and B). These gradients are assumed to represent climate, as the ELA is a function of both temperature and precipitation, at that elevation on the glacier (*16*). The steepened YD summer climate gradient south of 55°N is supported by our multi-proxy temperature reconstruction (fig. S1) and a published chironomid-based temperature reconstruction (*6*) but less so by the indicator plant species approach and associated high spatial resolution climate simulations (*7*). None of the temperature proxies support the step change in YD climate indicated by the ELA shift north of 55°N, suggesting a precipitation control. Plotting the ELAs as a function of longitude from the Pyrenees through the Alps shows a positive ELA gradient of 13 m per degree of longitude for the YD, which is not found for the present day (Fig. 4C). The zonal YD ELA gradient is neither reflected in the mean summer temperature dataset (fig. S1) nor by other data and modeling (*6*, *7*), again indicating a precipitation control.

### Palaeoprecipitation

The ELA palaeoprecipitation (PPP_ELA_ and PPA_ELA_; Figs. 2 and 3) provides a unique regional perspective into the YD glacier-climate and associated atmospheric circulation. The positive northward ELA gradient, north of 55°N (Fig. 4, A and B), reflects an increasingly arid climate, which indicates a reduced number and/or intensity of storms and the presence of permanent and seasonal sea ice in the North Atlantic (Greenland, Iceland, and Norwegian Seas). South of 55°N, along the Atlantic margin and across the Mediterranean, the YD climate was wetter, while across the North European Plain (NEP), the climate was drier (Figs. 2 and 3). In the eastern Mediterranean, notably high-precipitation regions are centered over the Dinaric Alps and northern Turkey (Fig. 2). The magnitude of the PPA_ELA_ anomalies over these two regions (Fig. 3) is, to some degree, a function of the station density in the modern gridded climatology. Attempts have been made to improve the anomalies over the Dinaric Alps, but this was not possible for Turkey. For both these regions, the PPP_ELA_ provides the best characterization for the YD climate (see Materials and Methods). Assuming that the PPP_ELA_ and PPA_ELA_ patterns reflect air mass advection at synoptic to sub-synoptic scales, i.e., storm tracks, they can be used to assess the prevailing YD atmospheric circulation across Europe.

## DISCUSSION

### Atmospheric circulation

The Laurentide ice sheet was still large enough to affect the hemispheric atmospheric circulation during the YD (*4*), as at the Last Glacial Maximum (LGM) (*13*, *14*). In the North Atlantic, the permanent and winter sea ice fronts were located substantially farther south than present-day (*7*, *8*, *10*, *19*), shifting storm track trajectories farther south than present-day (*12*). The FIS (*20*) (Figs. 1 to 3) provided a topographic barrier to eastward atmospheric flow (*7*, *13*, *14*, *21*), and the NEP was a permafrost environment (cold and arid) (*22*). Figures 2 and 3 suggest that, combined, the FIS and NEP generated a blocking system, a combination of a topographic barrier and a high-pressure region (FIS-NEP^HP^), which directed storm tracks along the western Atlantic margin (WAM; the British Isles, France, and the Iberian Peninsula) and into the Mediterranean. Following a circulation weather type approach, the YD precipitation pattern most closely resembles the modern Scandinavian (m-SCAND) circulation, although here shifted further south and formed under different boundary conditions. m-SCAND, predominantly a winter circulation (*23*), generates increased storm tracks along the WAM during October to March (*24*) with temporally and spatially distinct precipitation patterns across the autumn, winter, and spring (fig. S2). During the autumn and early part of winter (September through November), precipitation increases over the WAM and western Mediterranean. For mid-winter (December to February), precipitation is higher over the Iberian Peninsula and across much of the Mediterranean. In late winter to early spring (March through May) (the end/beginning of the accumulation/ablation season), the circulation returns to that resembling the early winter configuration. The FIS-NEP^HP^ is also likely to have been present during the summer (*7*), meaning that the palaeo-SCAND (p-SCAND) circulation may have been persistent throughout much of the year.

The increased/positive precipitation/anomaly YD pattern in the eastern Mediterranean is interpreted to be the result of increased strength and/or southward shift of the west-east zonal flow during mid-winter (Fig. 2) compared to modern day (Fig. 3 and fig. S2). In addition, cyclonic atmospheric wave breaking, associated with a southerly displaced PFJS (*14*, *18*, *25*) during winter, would have increased the outbreaks of cold northerly air over the relatively warm Mediterranean, providing ideal conditions for synoptic and mesoscale cyclogenesis (*18*, *26*). SSTs cooled relatively less in the eastern than the western Mediterranean during the YD (*27*, *28*), similar to the LGM (*29*), concomitantly enhancing cyclogenesis. The p-SCAND circulation pattern, similar to m-SCAND but more southerly displaced, provides a better explanation for the YD precipitation anomalies than a persistent negative phase North Atlantic Oscillation, which has been suggested for the LGM (*30*) and would enhance precipitation only across the Iberian Peninsula and Mediterranean.

The atmospheric circulation elucidated by this study (Figs. 2 and 3) is in general agreement with that found in most numerical modeling experiments for the LGM (*13*, *14*, *18*) and YD (*7*). The PPP_ELA_ (Fig. 2) is determined using a high spatial resolution digital elevation model (DEM), so topography is more realistic than the smoothed landscapes used in numerical modeling experiments [and modern gridded climate (*31*)], generally resulting in PPP_ELA_ values being larger than those produced by numerical models for the LGM (*13*) and YD (see Materials and Methods) (*4*, *8*, *32*–*35*). It is important to remember that the ELA_thl_, and accordingly the PPP_ELA_, is theoretical in areas where glaciers did not exist. It is accordingly problematic to directly compare the quantitative results from this work with those from climate model simulations. In addition, mesoscale cyclogenesis, which is not resolved in climate model simulations, is known to affect ELAs (*36*). The most marked example of this is the notable YD ELA depression identified in the Dinaric Alps (Fig. 1), which is in line with previously reported, empirically derived palaeo-ELA depressions (*37*) and the present-day precipitation pattern (*31*).

Despite the caveats noted above, climate model simulations providing total annual precipitation estimates for the YD are available [e.g., (*4*, *8*, *32*–*35*)] (fig. S3), and it is of value to compare the patterns of palaeoprecipitation generated by numerical models and that generated from the palaeoglacier ELAs (PPP_ELA_) (Fig. 2). The models are in general agreement regarding the pattern around the Mediterranean. Precipitation increases with latitude, from North Africa to the Iberian Peninsula (approximately 43°N), and is constant in longitude. The magnitude of the northward increasing precipitation gradient varies from 100 to 1000 mm a^−1^ (fig. S3E) to 100 to 600 mm a^−1^ (fig. S3, B and C). A shallower precipitation gradient (400 to 600 mm a^−1^) with two higher-precipitation centers over Corsica–Northwest Italy and the eastern Mediterranean just west of Crete is evident in (*32*) (fig. S3A). For the Mediterranean region, the pattern shown in fig. S3A is similar to PPP_ELA_ (Fig. 2), with precipitation increasing northward from North Africa to a high at approximately 40°N over the Iberian Peninsula before declining to the north over the remainder of the Iberian Peninsula. Precipitation generally increases eastward across the Mediterranean, and the two precipitation highs in Fig. 2 are farther east than in fig. S3A, centered on the mountains of the Dinaric Alps and northern Turkey. Precipitation highs are evident over the Dinaric Alps and Maritime Alps in (*35*) (fig. S3F), but only the former is seen in the PPP_ELA_ (Fig. 2). In general, the magnitude of the total annual precipitation varies between the climate models and is lower than PPP_ELA_ (Fig. 2), for the reasons noted above, except for (*35*) (fig. S3F), which has greater agreement with PPP_ELA_ over the Dinaric Alps and the Northern Iberian Peninsula.

Farther north, the climate models generally agree with each other and this study, that precipitation is lower across the NEP (Fig. 2 and fig. S3). Likewise, the climate models are in general agreement that along the western seaboard of Europe, there is a northward decline in precipitation. In some models, the decline starts from ~45°N (fig. S3, C to E), while in others, there is a precipitation high centered over, or west of, the British Isles (fig. S3, B and D to F). The latter pattern is in closer agreement with the results presented here (Fig. 2). None of the climate models identify the change in glacier climate, centered around 55°N, identified by the palaeo-ELA and palaeoprecipitation data (Figs. 1 to 4), taken here to represent the mean location of the PFJS during the first half of the YD.

Other proxy records that assess the hydroclimate, e.g., speleothems, could potentially provide another routeway for cross-correlation of the annual precipitation reconstructions, but for the most part, they do not generate quantitative palaeoprecipitation estimates, and no regional dataset is available. One site that is potentially useful is La Garma cave [85 meters above sea level (masl)] in Cantabria, Spain, where YD precipitation is estimated to be 1050 mm a^−1^ (*38*). It is located approximately 130 km from the nearest palaeoglacier site (Peña Vieja), which has a palaeo-ELA at 1886 masl and PPP_ELA_ of 2407 mm a^−1^. This gives a YD vertical precipitation gradient of 76 mm/100 m (7.25%/100 m) for the 1785 m elevation difference across the 130 km between the sites. An estimate for the modern precipitation gradient, using data from (*39*), is 18 mm/100 m. Precipitation gradients are notoriously variable, but the palaeoprecipitation gradient does not seem unreasonable compared to some modern values; for example, in Switzerland, they range between 23 and 158 mm/100 m (*40*); in Slovenia, 97 and 453 mm/100 m (*41*); and in Norway, 15%/100 m (*42*).

This study represents the first palaeoglacier-based investigation of the atmospheric circulation during the YD across Europe and is the largest of such study, for any period, during the last glacial cycle. The PPP_ELA_ (Fig. 2) and PPA_ELA_ (Fig. 3) patterns provide a new insight into the atmospheric circulation over Europe during the first half of the YD. It is best explained as a p-SCAND circulation, which was likely persistent across much of the year. Overall, the FIS and the NEP provide a topographic and atmospheric high-pressure blocking system, which, in combination with the extensive sea ice in the North Atlantic, pushes the storm tracks and the PFJS southward, although not as far south as indicated in LGM climate simulations (*13*, *14*). The topographic barrier provided by the ice sheet was permanent, but the influence of the migrating sea ice front and the magnitude of the high-pressure systems would have varied across the year, leading to seasonal variations in the circulation and precipitation pattern. Under p-SCAND, by analogy with the modern circulation, autumn and spring precipitation is interpreted to be higher along the WAM and in the western Mediterranean. p-SCAND winter precipitation is interpreted to be higher across the Iberian Peninsula and whole Mediterranean. Overall, the western seaboard of Europe, south of ~54°N to 55°N, much of the Iberian Peninsula, and the western and eastern Mediterranean were all wetter than the present-day, modeled LGM (*13*, *18*), and modeled YD (*4*, *8*, *32*–*35*), although comparisons between climate model outputs and Fig. 2 are not simple. The palaeoprecipitation estimates calculated here are compatible with hydroclimate estimates from speleothems (*38*) in terms of precipitation gradients. The wetter climate identified in this study stems from enhanced cyclogenesis at both synoptic and mesoscales. Reconstructed palaeoglacier ELAs provide a regional perspective on YD atmospheric circulation and palaeoprecipitation currently not available using other proxies and generate much needed data, which may be used to tune/validate numerical modeling experiments (*17*).

## MATERIALS AND METHODS

### Regional assay

Extensive mining of the published literature was undertaken to identify palaeoglacier sites, within the study area, where lateral/frontal/fronto-lateral moraines had been dated to, or within error of, the YD. The interpretation of the dates provided in the original paper were followed, unless recalibrations/recalculations (see below) placed the site outside of the YD time window or sites did not meet some of the other assessment criteria. For a dated moraine to be used for palaeoglacier reconstruction, it also had to either appear on a map with coordinates or be readily identifiable on available satellite/aerial imagery. On the basis of the original paper/s and an assessment of free-to-access imagery, any glacier that appeared to have had the possibility of any significant mass loss from calving was excluded. Sites were then assessed for the fidelity of the chronology, by review of the dating context, the landform and sample/s dated, and the dating technique. All relevant information was captured in a database to facilitate subsequent recalibration using IntCa13 for ^14^C and recalculation for cosmogenic ^10^Be, involving, where applicable, new production rates, accelerator mass spectrometry standards, and half-life revisions using the atoms per gram data published in the original papers (data file S1). Where these were not available, they were requested from the laboratory that undertook the analyses. This rigorous quality control allowed maximization of the number of sites that were dated either directly or by morphostratigraphical relationship to the YD. Note that the techniques used to constrain the ages of the moraines have a range of uncertainties (typically in years, ^14^C ± 120, ^10^Be and ^36^Cl ± 1000, ^26^Al ± 1500, and U/Th ± 300). Although these uncertainties are quite large for cosmogenics, the target was moraines that are formed during readvances/stillstands, i.e., during climatic periods favorable for glacier existence/stability. Provided the age range for the moraines overlapped with the time window of the YD, here taken to be 12.9 to 11.7 ka before the present (B.P.), they were selected as sites for palaeoglacier reconstruction. The moraines meeting all our quality controls were accepted as YD and were used in subsequent workflows, providing a final dataset of 122 palaeoglaciers (Fig. 1 and data file S2). Because of the uncertainties associated with cosmogenic exposure ages used for chronologically constraining most of the glaciers, the regional picture is taken to represent a snapshot of YD maximum glaciation, where the maxima are considered synchronous. As the first half of the YD is the coldest and most stable period, this maxima snapshot is assumed to have occurred within this time window. This is deemed reasonable as the glaciers investigated should all have response times significantly less than 500 years.

### Glacier reconstructions

To remove any potential bias/errors in the reconstructed palaeoglaciers from the original papers, an equilibrium profile modeling approach was applied. Glacier thickness is reconstructed along a central flowline, assuming perfect plasticity (Eq. 1)

$$\tau_{y}=\tau_{d}=\rho \mathrm{gH}\frac{\delta h}{\delta x}$$ (1)

where τ_y_ is the yield stress, τ_d_ is the driving stress, ρ is the density of ice, *g* is gravity, *H* is ice thickness, and $\frac{\delta h}{\delta x}$ is the ice surface slope. This is then solved iteratively, step by step, up-glacier from the frontal moraine (Eq. 2) (*43*, *44*)

$$h_{i+1}^{2}-h_{i+1}(b_{i}+b_{i+1})+h_{i}(b_{i+1}-H_{i})-\frac{2\tau_{\text{av}}\Delta x}{F\rho g}$$ (2)

where *h* is the ice surface elevation; *b* is the bed elevation; τ_av_ is the average basal shear stress; Δ*x* is the step length; *F* is the shape factor; ρ, *g*, and *H* as above; and *i* is the iteration (step) number. The shape factor *F* takes account of the lateral drag imposed by the constraining topography, should it exist, i.e., it is small if the valley is narrow and deep, and high if it is wide and shallow, reaching 1 for unconstrained ice masses, e.g., plateau ice fields

$$F=\frac{A}{\mathrm{Hp}}$$ (3)

where *A* is the glacier cross-sectional area and *p* the perimeter length of the cross section (equivalent to the wetted perimeter in a river cross section). *F* is calculated automatically or at used defined intervals along the length of the glacier flowline (*45*). The equilibrium profile approach requires no a priori forcing via mass balance, which allows the accumulation and thus precipitation to be estimated subsequently. This approach is preferred over the use of dynamic models where mass balance forcing is used to generate multiple glaciers that fit the mapped moraine under a suite of climate scenarios, i.e., temperature-precipitation envelopes. A bespoke Python-coded toolbox was used to rapidly implement the solution to Eqs. 2 and 3 in ArcGIS (*45*). Full details of the tool and its operation can be found in (*45*), but in brief, the tool calculates the ice thickness, point by point, up-glacier from the frontal moraine along a flowline at a user-defined step interval. For geometrically simple valley and cirque glaciers, a uniform basal shear stress of 100 kPa (*46*) was applied along the length of the glacier. For ice masses with low bed slope angles near the ice divide, the shear stress was reduced accordingly. Shape factors (*F*) were applied where glacier flow was topographically constrained. The centerline ice surface elevation generated from Eq. 2 is propagated outward, perpendicular to the flowline, at a user-defined interval, to generate an extensive point cloud for the reconstructed ice surface. A raster digital elevation model is generated from the point cloud, using kriging interpolation, clipped by the topography, and, where applicable, user-defined ice divides, generating the full 3D glacier surface (*45*). For more complex geometries, multiple second- and third-order tributaries may be added to provide a more robust reconstruction. Because of the relatively limited range of driving stresses generated by mountain glaciers, averaged along their length, the reconstructed ice surface elevations are expected to be reasonable approximations of reality. To test the approach outlined above, the ice thickness of an extant ice mass needs to be known along the glacier length. Ice thickness data for Folgefonna ice cap in southern Norway and Griesgletscher in the Swiss Alps have previously been used to assess the approach (*45*). The results were very encouraging for the parameters used in this study; ice surfaces were generated using kriging, and shape factors were calculated at user-defined cross sections. For Folgefonna, the volume and area differences were −0.22 km^3^ (−8%) and −2.53 km^2^ (−9%), and for Griesgletscher, they were 0.014 km^3^ (10%) and −0.44 km^2^ (−8%).

### Equilibrium line altitudes

Glaciers can be linked to climate through the ELA (*16*), and the ELA can be readily calculated for the reconstructed 3D glacier surfaces (*45*, *47*). Multiple ratio-based approaches for determining the glacier ELA are available. Because of the size of the dataset used here, with attendant variation in glacier types, which encompasses cirques, valley glaciers, and icefields, the Area Altitude Balance Ratio (AABR) is chosen (*47*, *48*). This method takes account of glacier hypsometry and is the best ratio-based method for ELA determination in general and especially where a wide range of glacier types and geometries are involved (*48*). In addition, the global AABR (*48*) was determined from glaciers spanning a range of modern climates, which should, as far as possible, encompass the range of climates experienced by the reconstructed glaciers. An AABR of 1.7 was chosen (*48*) and applied to all the reconstructed glaciers. It is important to emphasize here that using the AABR to calculate the palaeoglacier ELA does not lead to circular reasoning later in the workflow, specifically in regard to the calculation of the palaeoprecipitation, as the method is based on geometrical moments. Full details of the AABR method can be found in (*48*) but are briefly provided here

$$\text{AABR}=\frac{b_{\text{nab}}}{b_{\text{nac}}}=\frac{{\bar{z}}_{\text{ac}}A_{\text{ac}}}{{\bar{z}}_{\text{ab}}A_{\text{ab}}}$$ (4)

where ac and ab refer to the accumulation and ablation areas, respectively; *b*_n_ is the net mass balance gradient; $\bar{z}$ is the area-weighted mean altitude; and *A* is area. The ELA for each glacier is calculated in ArcGIS using a bespoke tool (*47*), which uses the AABR (1.7) and applies the summing of moments to contour elevation intervals. The ELA for the chosen ratio is determined as the mid-elevation of the contour interval where the overall net balance changes sign from positive to negative (calculating upward from the frontal moraine) (*47*). These parameters and workflow were then used to generate an ELA for 3D surface reconstructions for each of the 122 palaeoglaciers (data file S2).

It has been previously suggested that the ELA uncertainty using the Accumulation Area Ratio approach for reconstructed glaciers is likely to be ±50 m for smaller and geometrically simple glaciers rising to ±100 m for larger and more complex glaciers (*18*). Applying a free-air lapse rate of 0.0065°C m^−1^ [International Standard Atmosphere (ISO) 2533:1975], these errors equate to a ±0.325°C and ±0.65°C temperature difference, respectively, at the ELA. The numerical approach used here for the glacier reconstructions and application of the AABR for ELA calculation, which accounts for the glacier hypsometry associated with both simple and complex glacier geometries, is believed to have reduced the overall error. Taking the two test glaciers from above and using an AABR of 1.7, the ELA for Folgefonna was determined to be +3 m from that estimated for the extant icefield surface (*45*). Similarly, for Griesgletscher, the difference was determined to be −2 m from that estimated for the extant glacier (*45*). Using the same ABBR, ELAs were calculated for satellite-derived DEMs, for the dataset used in (*48*), and compared with the measured ELAs. This suggested that the uncertainty is much closer to ±50 m, as suggested in (*18*), but for all glacier sizes.

Plotting the ELAs along western Europe as a function of latitude is a simplifying approach to allow the climatic transition (breakpoint) associated with the PFJS to be identified. The identification of the breakpoint used a piecewise regression for the south-north ELA transect (fig. S3, A and B), in R, following two approaches. The first implemented an iterative searching approach, within a user-defined range encompassing the breakpoint, and identified the combination that returned the lowest mean squared error. Thus, the two regressions are not forced to touch or to be continuous and returned the breakpoint at 55.02°N. The second approach used the segmented package in R, and here, the segments are continuous and touching. This approach located the breakpoint at 54.66°N. These values should not be taken as an absolute location of the PFJS during the first half of the YD. Rather, this is the location of the transition zone south of which there is a significant maritime influence and substantial total annual precipitation, north of which the environment becomes polar with less total annual precipitation.

In order to generate a regional perspective on ELAs, and subsequently climate, during the YD the full dataset of reconstructed ELAs was then gridded to generate a spatial representation of theoretical ELAs (ELA_thl_). They are theoretical because a true ELA will only exist where there is sufficient topography above the ELA_thl_, for a glacier to form (Fig. 1).

### Palaeotemperature reconstruction

The ELA is the point on a glacier where, at the end of the mass balance year, the surface accumulation, controlled by precipitation, equals the surface ablation, controlled by temperature. The two variables have been linked via empirically derived relationships measured at the ELA, i.e., the total annual precipitation versus the mean summer air temperature (MSAT_ELA_) where summer equates to June, July, and August (*16*). To determine palaeoprecipitation at the ELA, the MSAT_ELA_ is required. To a first order, mean monthly air temperatures can be assumed to follow a sinusoid across the year. At the ELA, the MSAT can then be derived by fitting a sinusoid through any two of the mean annual air temperature (MAAT), the mean temperature of the coldest month (MTCM), or the mean temperature of the warmest month (MTWM). YD palaeotemperatures for the study area are available from multiple proxies. These may be terrestrial, providing estimates for summer temperature (e.g., MTWM from chironomids, pollen, and coleoptera), MAAT (permafrost/ice wedge casts), or MTCM (ice wedge casts) (*49*). Marine data can provide SSTs derived from alkenones, diatoms, dinocysts, foraminifera, and lipid markers. The errors on the temperature proxies, typically used for regional-scale temperature reconstructions (*6*, *7*), are between ±1° and ±2.5°C: for terrestrial summer temperatures, a root mean square error of prediction (RMSEP) of 1.4°C for European chironomids, ±0.83°C for coleoptera mutual climate range (*50*), and 0.64° to 1.25°C for pollen (*51*); while for SST, an RMSEP of ~1°C for diatoms (*52*) and ~0.5° to 1.5°C for foraminifer using transfer functions (*53*); and ±0.83°C for Mg/Ca ratios (*54*), ±1.1°C alkenones black (*55*), and ±2.5°C for lipids (*56*).

In total, the proxy palaeotemperature data have been mined from the published literature, generating a dataset of 132 sites across Europe (data file S3). We did not use the YD temperature reconstruction in (*7*) into our dataset because it provided a mean for the whole of the YD and not the first half, as we required. The temperature estimates provided in the original publications were used directly, except for some chironomid temperatures, which had been recalculated in (*6*), and these are indicated (data file S3, column G). Where necessary terrestrial proxy temperatures were extrapolated to SLE temperatures by the application of a 0.0065°C m^−1^ lapse rate (ISO 2533:1975). The chronologies for the different proxies are variable, in some instances limited to a single YD record, while in others, a well-constrained age-depth model is available. To maximize the number and spatial coverage of data points available across the region, as far as possible, we obtained (or generated) a single (mean) value temperature for the first 500 years of the YD, i.e., from 12,900 to 12,400 B.P. Where a single value only was available, this was used, and where none were available for the first 500 years, the nearest one, within the YD, was selected. The chronological data in the original publications were used as reported, and the calculation of a mean temperature for the 500-year time window, for the first half of the YD, limiting any potential issues with age range errors. We chose the first half of the YD because the glacier chronologies are limited by the uncertainties associated with cosmogenic exposure ages, this is generally viewed as the coldest and most stable part (*12*), and the time window of 500 years should be significantly greater than the response time of any of the palaeoglaciers.

MAAT_SLE_ was derived from marine SST proxies in the Mediterranean region and from periglacial landforms for Central and Northern Europe and generated 35 data points (data file S4). The data showed a strong north-south trend, as reported elsewhere (*22*), so during the gridding process (kriging), anisotropy of 180° was applied to account for this. The resulting dataset provided a gridded MAAT_SLE_ between 31°40′N and 56°41′N (fig. S1).

The MTWM_SLE_ dataset was derived using chironomids, coleoptera, diatoms, foraminifera, and pollen and generated 92 data points (data file S5). First-degree detrending was applied, and it was then interpolated using kriging. The resulting dataset provided a gridded MTWM_SLE_ between 40°49′N and 71°8′N (fig. S1).

Ideally, the two gridded temperature datasets should have the same spatial coverage as the palaeoglaciers. The MAAT_SLE_ dataset only reached 56°41′N, and the dataset covered neither the Moroccan Atlas Mountains nor the easternmost part of Turkey. To accommodate for this, the following procedures were undertaken to extend the gridded temperature datasets to the outer ranges of the spatial extent of the palaeoglacier ELA reconstructions.

1) For Scotland, a second-order polynomial was fitted through the MAAT_SLE_, calculated at the locations of palaeoglacier sites in the British Isles located south of 56°41′N. This produced a very strong [*R*^2^ (coefficient of determination) = 0.997] negative northward correlation (fig. S4).

2) For the sites in Norway, a second-order polynomial was fitted through the SLE MAAT_SLE_ from the full dataset provided in data file S4. This produced a very strong (*R*^2^ = 0.959) negative northward correlation (fig. S5).

3) To generate the MTWM_SLE_ for the area south of 40°49′N, a second-order polynomial was fitted through the dataset (data file S5). This produced a very strong (*R*^2^ = 0.753) negative northward correlation (fig. S6), generating the southward projection of MTWM_SLE_ for palaeoglacier sites in Spain, Morocco, Greece, and Turkey.

The MSAT_SLE_ was determined from the MAAT_SLE_ and the MTWM_SLE_ by fitting a sine curve through these three points (fig. S7) and taking the mean for June, July, and August temperatures. For the final part of the workflow, the MSAT_ELA_ was then determined from the MSAT_SLE_ by applying the 0.0065°C m^−1^ lapse rate for all the palaeoglaciers.

### Palaeoprecipitation

A reanalysis of the dataset of Ohmura *et al.* (*16*) was undertaken, and as originally, the MSAT_ELA_ was related to the total annual precipitation at the ELA. Our reanalysis generated a slightly better fit to the data (correlation coefficient of 0.90273) than the original paper, and the resulting equation for the potential total annual palaeoprecipitation (snow and rain) at the ELA (PPP_ELA_) in millimeters water equivalent is

$${\text{PPP}}_{\text{ELA}}=691.83+294.31T+7.7171\;T^{2}$$ (5)

where *T* is the MSAT_ELA_ in °C.

The PPP_ELA_ data points were interpolated, as above (kriging), to generate a regional map of YD potential palaeoprecipitation, and it must be remembered that this is precipitation at the glacier ELA (Fig. 2). As noted above, proxy-derived temperatures were taken as they appeared in the original publication [except from some chironomid data recalculated by (*6*)], but it is acknowledged these come with some uncertainties. The impact of these uncertainties was assessed assuming for the MTWM ±1.5°C and the MTCM ~±5°C. For most cases, this resulted in a change of <10% in the PPP_ELA_ with a maximum of ~20% for the worst-case scenario. However, for all these scenarios, if they are applied consistently across the dataset, the PPP_ELA_ pattern remains essentially the same. In addition, MTWM determined by the plant indicator species (*7*), which tends to provide a systematically higher estimate, has not been incorporated into our dataset. Its inclusion would have increased the MTWM, resulting in increased estimates of PPP_ELA_ on the order of ~5 to 10% but, again, would not have significantly changed the regional pattern.

To better contextualize the pattern, potential palaeoprecipitation at the ELA standard anomalies (PPA_ELA_) have been calculated using

$${\text{PPA}}_{\text{ELA}}=\frac{{\text{PPP}}_{\text{ELA}}-\bar{x}}{\sigma }$$ (6)

where $\bar{x}$ is the mean, and σ is the SD of the total annual precipitation (both in millimeters) for the period 1950 to 2000 (although some regions have incomplete time series, e.g., in the Atlas and Tatra Mountains) (*39*). Data from (*39*) provides a Europe-wide total annual precipitation on a 1-km gridded raster. It incorporates data from WorldClim (*57*) and E-OBS (*31*) and best represented the extreme precipitation values, found in coastal SW Norway, W Scotland, and Montenegro. That said, it is also acknowledged that “the extremes of the downscaled data are most likely too conservative” (*39*). An underestimation of present-day precipitation, especially in the high mountains, would lead to an overestimation of PPA_ELA_. “This downscaling method still suffers from the lack of weather station density inherited from both input datasets” (*39*). This issue has affected the PPA_ELA_ calculated for the Dinaric Alps, specifically over Montenegro, which is a region of extreme precipitation gradients in the present day. The data from (*39*) indicate modern total annual precipitation of ~1000 mm while station data from (*58*) reports 4593 mm at 937-m elevation (Crkvice station), 9 km SW from the reconstructed glacier Gjorni Do. The station data from (*58*) have been incorporated into the PPA_ELA_ calculation (Fig. 3), but this has not solved the issue entirely. For example, in the Dinaric Alps, local-scale modern precipitation is known to exceed that indicated by the PPP_ELA_ reconstructed in Fig. 2 (*37*). It is cautioned therefore that the very high positive precipitation anomalies, especially over the Dinaric Alps and Turkey, are overestimates of the true PPA_ELA_. For these two regions, the PPP_ELA_ provides a better characterization of the YD climate. Additional complications arise due to the difference in grid resolution between the modern precipitation data at 1 km (*39*) and the DEMs that were used to undertake the glacier reconstructions, 30 m or less. This means that elevation in the modern precipitation dataset (*39*) is smoothed and reduced in comparison to the glacier reconstruction DEMs. To address this, the following approach was taken. If the elevation of the grid cell in the 1-km DEM lies within ±100 m of the palaeoglacier ELA, then that value was taken for present-day precipitation. If this was not the case, the search was expanded out by one grid cell in all directions, and the one lying within ±100 m and closest in elevation to the ELA was chosen. If necessary, the search was expanded out to the next cell, i.e., two grid cells in all directions, and this sufficed for all sites.

## Supplementary Material

http://advances.sciencemag.org/cgi/content/full/6/50/eaba4844/DC1

Data file S1

Data file S2

Data file S3

Data file S4

Data file S5

Adobe PDF - aba4844_SM.pdf

Atmospheric circulation over Europe during the Younger Dryas

## SUPPLEMENTARY MATERIALS

Supplementary material for this article is available at http://advances.sciencemag.org/cgi/content/full/6/50/eaba4844/DC1
