# Supplementary material for: Atmospheric circulation over Europe during the Younger Dryas
Source: Sci Adv. 2020 Dec 11;6(50):eaba4844. doi: 10.1126/sciadv.aba4844 (PMC7732201; doi:10.1126/sciadv.aba4844)
Supplement: http://advances.sciencemag.org/cgi/content/full/6/50/eaba4844/DC1 [file supp_6_50_eaba4844__index.html]

Science Advances | Science AdvancesAAASSearchScience AdvancesMenu

## Supplementary Materials

# Atmospheric circulation over Europe during the Younger Dryas

Brice R. Rea, Ramón Pellitero, Matteo Spagnolo, Philip Hughes, Susan Ivy-Ochs, Hans Renssen, Adriano Ribolini, Jostein Bakke, Sven Lukas, Roger J. Braithwaite

Download Supplement

**The PDF file includes:**

- Figs. S1 to S7
- Legends for data files S1 to S5
- References

**Other Supplementary Material for this manuscript includes the following:**

- Data file S1
- Data file S2
- Data file S3
- Data file S4
- Data file S5

**Files in this Data Supplement:**

- Adobe PDF - aba4844\_SM.pdf
- aba4844\_Data\_file\_S1.xlsx
- aba4844\_Data\_file\_S2.xlsx
- aba4844\_Data\_file\_S3.xlsx
- aba4844\_Data\_file\_S4.xlsx
- aba4844\_Data\_file\_S5.xlsx
