# Supplementary material for: Atmospheric circulation over Europe during the Younger Dryas
Source: Sci Adv. 2020 Dec 11;6(50):eaba4844. doi: 10.1126/sciadv.aba4844 (PMC7732201; doi:10.1126/sciadv.aba4844)
Supplement: http://advances.sciencemag.org/cgi/content/full/6/50/eaba4844/DC1 [file supp_6_50_eaba4844__1.pdf]

Science Advances
AAAS

advances.sciencemag.org/cgi/content/full/6/50/eaba4844/DC1

Supplementary Materials for

Atmospheric circulation over Europe during the Younger Dryas

Brice R. Rea*, Ramón Pellitero, Matteo Spagnolo, Philip Hughes, Susan Ivy-Ochs, Hans Renssen, Adriano Ribolini, Jostein Bakke, Sven Lukas, Roger J. Braithwaite

*Corresponding author. Email: b.rea@abdn.ac.uk

Published 11 December 2020, *Sci. Adv.* **6**, eaba4844 (2020)
DOI: 10.1126/sciadv.aba4844

The PDF file includes:

Figs. S1 to S7
Legends for data files S1 to S5
References

Other Supplementary Material for this manuscript includes the following:

(available at advances.sciencemag.org/cgi/content/full/6/50/eaba4844/DC1)

Data files S1 to S5

Figs. S1 to S7

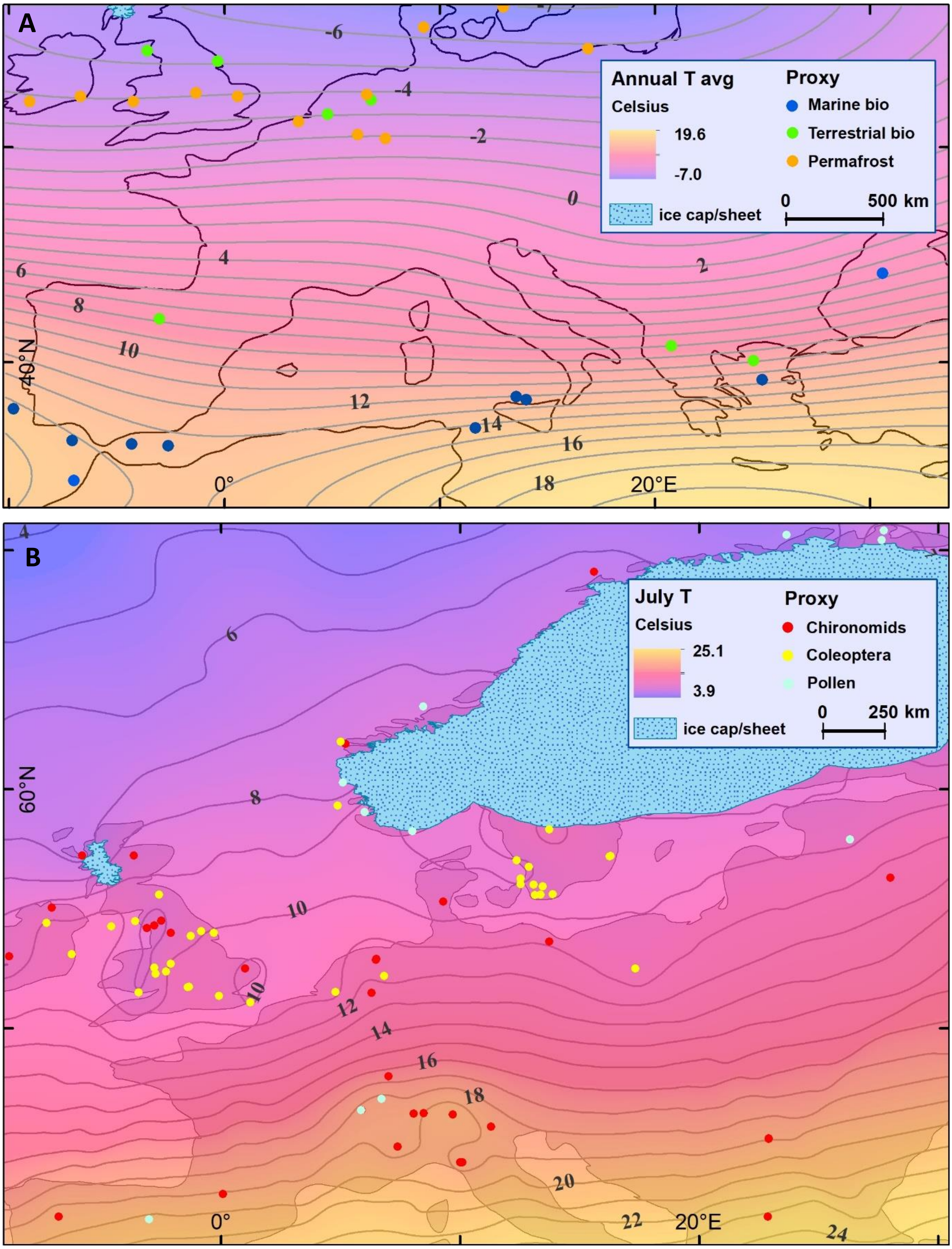

Fig. S1. Sea level equivalent Younger Dryas temperatures and palaeotemperature proxy sites. A, the mean annual air temperature (MAAT_{SLE}) and **B**, the mean summer (June, July, August) air temperature (MSAT_{SLE}). The proxy locations and types are indicated and colour coded. The SLEs are calculated using a free-air lapse rate of 0.0065 $°\text{Cm}^{-1}$. Both grids do not cover the entirety of the study area and so have been extended (see materials and methods).

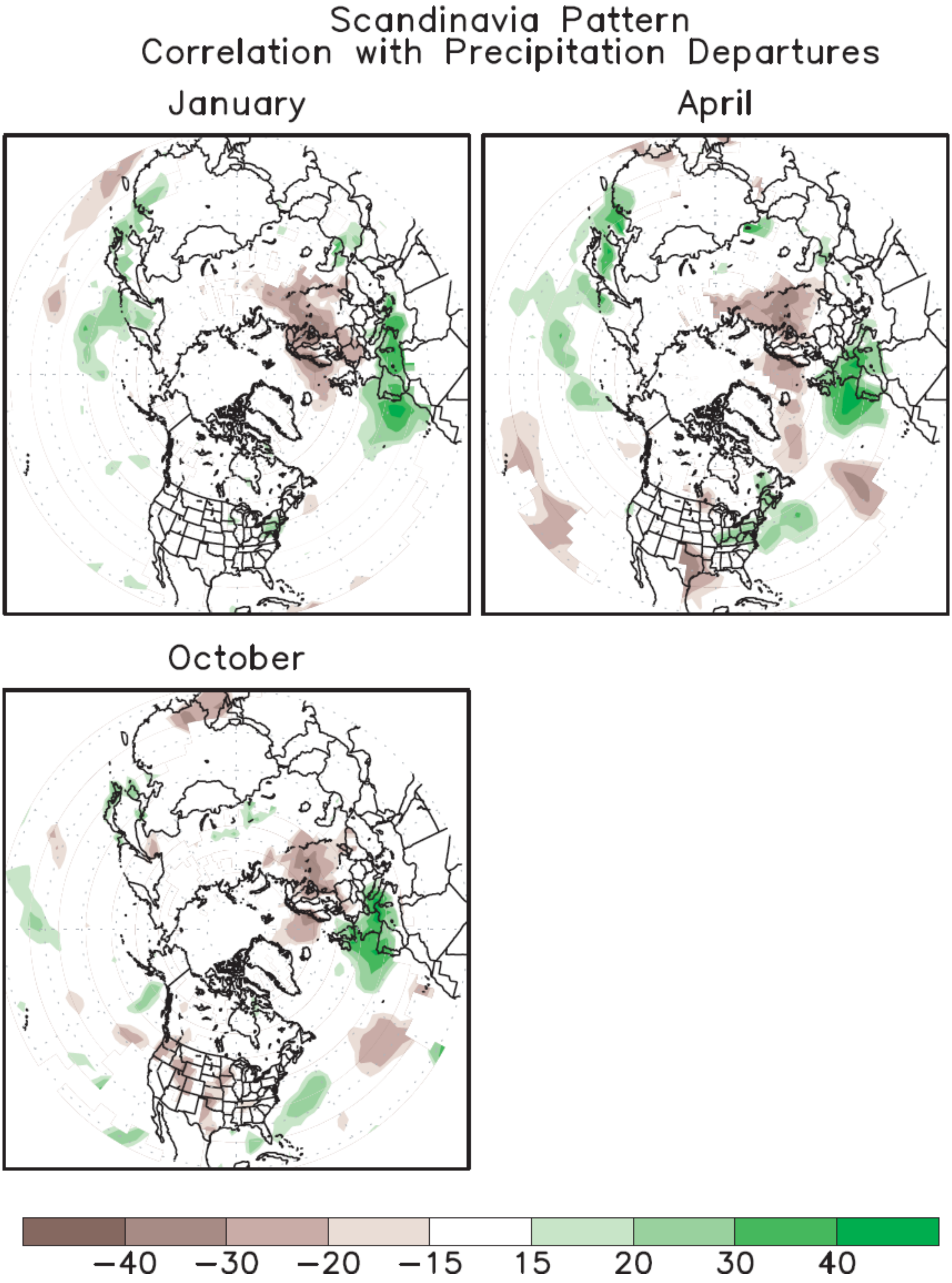

Fig. S2. Precipitation departures for the present-day positive phase SCAND circulation, for the period 1950-2000. Maps show the correlation between the SCAND index and precipitation departures for D-January-F, M-April-M, J-July-A and S-October-N windows. In mid-winter the storm tracks are guided zonally, across Iberia and the Mediterranean. In autumn and spring the storm tracks are constrained more to the western European margin and western Mediterranean. The figure was sourced from the NOAA, Climate Prediction Center at https://www.cpc.ncep.noaa.gov/data/teledoc/scand_pmap.shtml

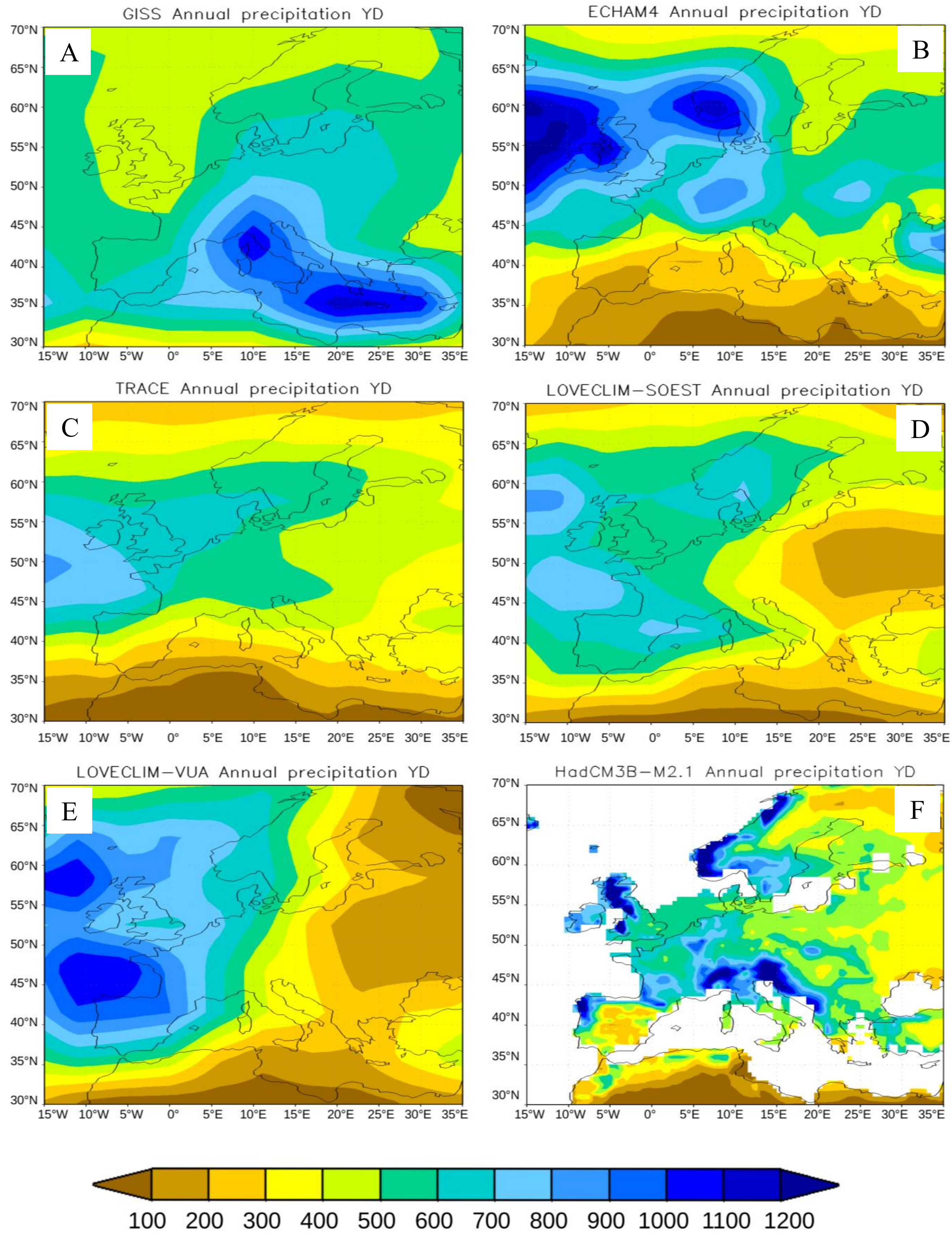
GISS Annual precipitation YD
A
ECHAM4 Annual precipitation YD
B
TRACE Annual precipitation YD
C
LOVECLIM-SOEST Annual precipitation YD
D
LOVECLIM-VUA Annual precipitation YD
E
HadCM3B-M2.1 Annual precipitation YD
F
70°N 65°N 60°N 55°N 50°N 45°N 40°N 35°N 30°N
15°W 10°W 5°W 0° 5°E 10°E 15°E 20°E 25°E 30°E 35°E
100 200 300 400 500 600 700 800 900 1000 1100 1200

Fig. S3. Mean YD precipitation estimates from multiple climate model simulations. The precipitation colour scale is in mm a^{-1} and the climate model used is indicated in the title bar: (**A)** is the GISS general circulation model (*32*); (**B)** is the ECHAM4 (European Centre/HAMburg) model (*22*); (**C)** is TraCE-21 (Transient simulation of Climate Evolutionof the last 21,000 years) using CCSM3 (Community Climate System Model version 3), (*33*); (**D**) is the LOVECLIM_SOEST (LOch–Vecode-Ecbilt-CLio-agIsm Model) model (*34*); (**E**) is the LOVECLIM_VUA model (*4*); (**F**) is the Bristol University version of HadCM3B-M2.1 and has been masked for the ocean (*35*).

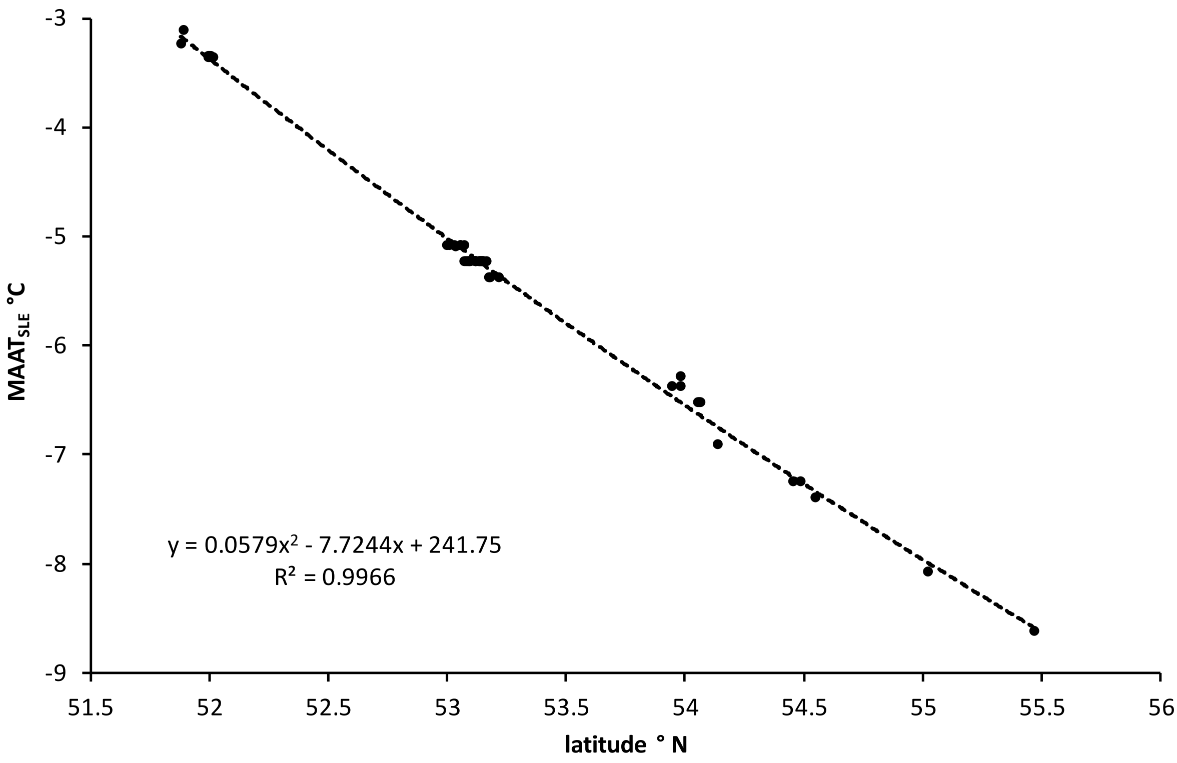

Fig. S4. Regression of sea level equivalent mean annual air temperature ($MAAT_{SLE}$) in °C on latitude, for YD palaeoglacier sites in the British Isles, south of 56° 41′ N. The second order polynomial was then used to project the $MAAT_{SLE}$ northwards for sites in Scotland north of 56° 41′ N, in order to facilitate calculation of the mean summer air temperature (see materials and methods).

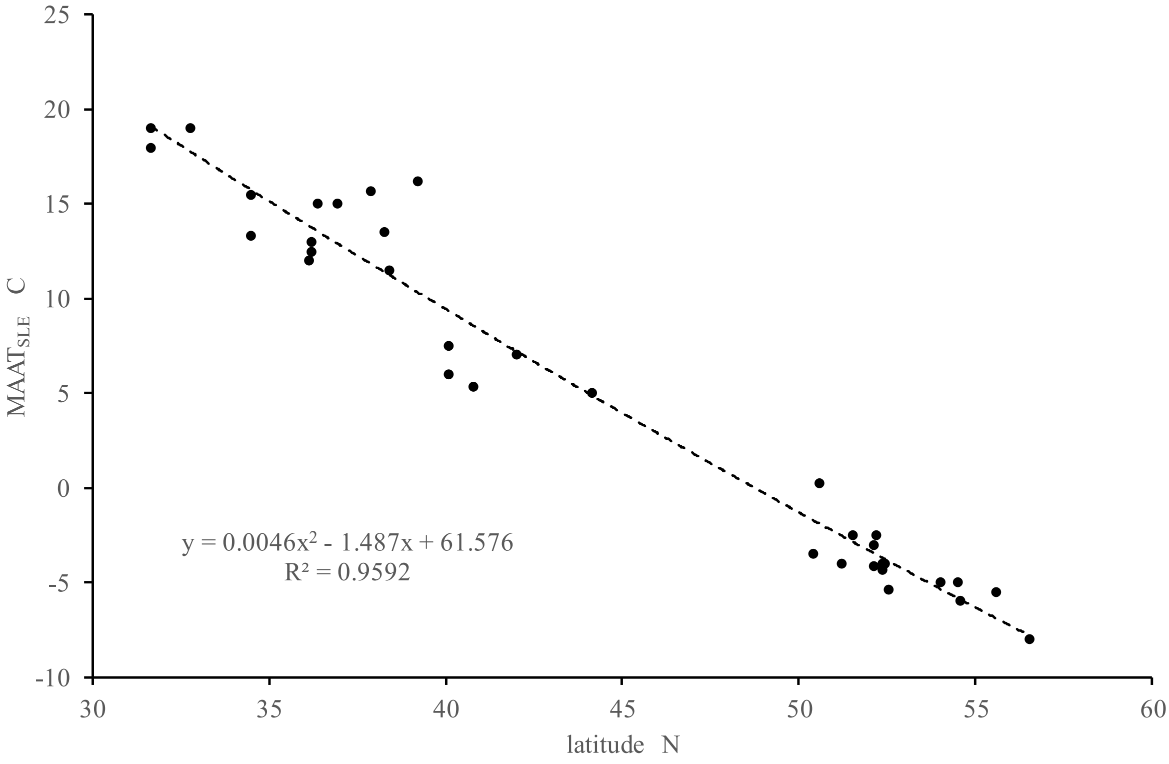

Fig. S5. Regression of sea level equivalent mean annual air temperature ($MAAT_{SLE}$) in °C on latitude, for the YD. The second order polynomial was then used to project the $MAAT_{SLE}$ northwards for sites in Norway, in order to facilitate calculation of the mean summer air temperature (see materials and methods).

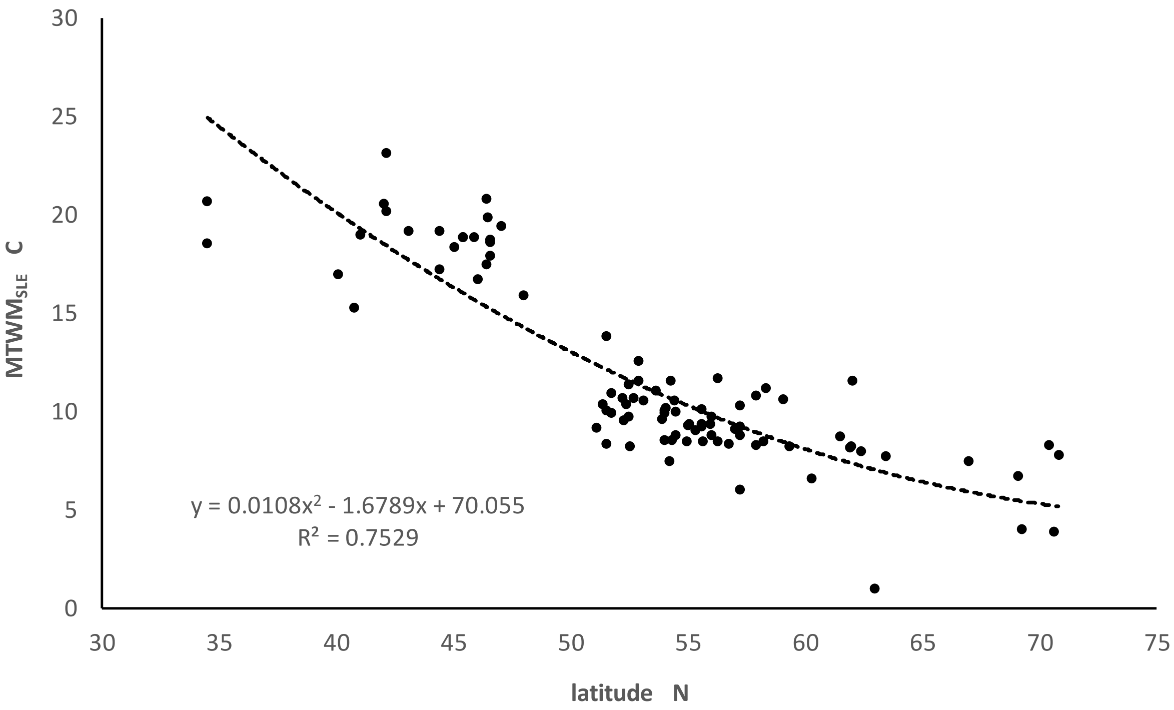

Fig. S6. Regression of sea level equivalent mean temperature of the warmest month ($MTWM_{SLE}$) in °C on latitude, for temperature proxy sites in Europe. The second order polynomial was then used to project the $MTWM_{SLE}$ south and east for sites in Spain, Morocco, Greece and Turkey, in order to facilitate calculation of the mean summer air temperature (see materials and methods).

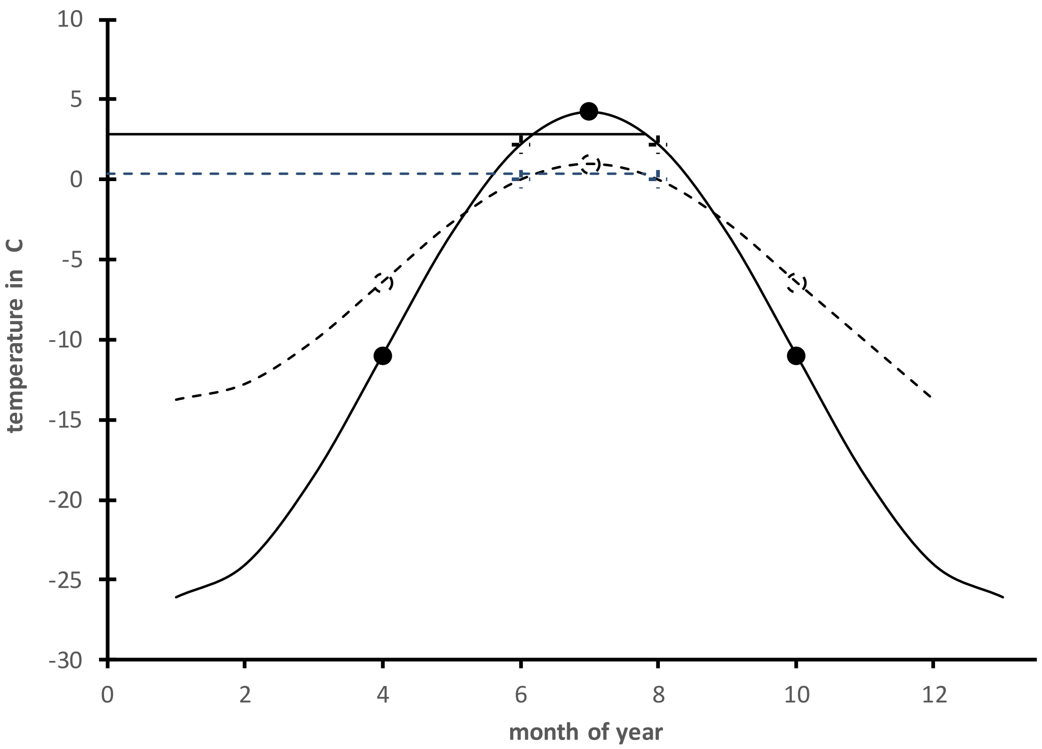

Fig. S7. Determination of, at the ELA, mean summer air temperature ($MSAT_{ELA}$) from the mean annual air temperature ($MAAT_{ELA}$) and the mean temperature of the warmest month ($MTWM_{ELA}$). In this example **t**he solid line is the fitted sinusoid for Piano del Praiet (ELA = 2335 m), and the dashed line is for Mosca (ELA = 2975). The filled and open circles are the $MAAT_{ELA}$ and $MTWM_{ELA}$ for Piano del Praiet and Mosca respectively. The crosses are the June and August temperatures used for calculation of the $MSAT_{ELA}$ which is shown by the solid and dashed horizontal lines.

Caption for Data files S1 to S5

Data_file_S1. List of the age control sites including any that were recalculated and the original source reference.

Data_file_S2. Full list of the reconstructed palaeoglaciers including the reconstructed ELA and PPP_{ELA}.

Data_file_S3. Full list of the temperature proxy data sites used to calculate the $MAAT_{SLE}$ and the $MTWM_{SLE}$ including reference to the original source.

Data_file_S4. Sites from Supplementary Data 3 used for the generation of the gridded $MAAT_{SLE}$.

Data_file_S5. Sites from Supplementary Data 3 used for the generation of the gridded $MTWM_{SLE}$.

REFERENCES AND NOTES

1. T. F. Stocker, D. G. Wright, Rapid transitions of the ocean's deep circulation induced by changes in the surface water fluxes. *Nature* **351**, 729–732 (1991).
2. R. B. Firestone, A. West, J. P. Kennett, L. Becker, T. E. Bunch, Z. S. Revay, P. H. Schultz, T. Belgya, D. J. Kennett, J. M. Erlandson, O. J. Dickenson, A. C. Goodyear, R. S. Harris, G. A. Howard, J. B. Kloosterman, P. Lechler, P. A. Mayewski, J. Montgomery, R. Poreda, T. Darrah, S. S. Q. Hee, A. R. Smith, A. Stich, W. Topping, J. H. Wittke, W. S. Wolbach, Evidence for an extraterrestrial impact 12,900 years ago that contributed to the megafaunal extinctions and the Younger Dryas cooling. *Proc. Nat. Acad. Sci. U.S.A.* **104**, 16016–16021 (2007).
3. C. Wunsch, Abrupt climate change: An alternative view. *Quatern. Res.* **65**, 191–203 (2006).
4. H. Renssen, A. Mairesse, H. Goosse, P. Mathiot, O. Heiri, D. M. Roche, K. H. Nisancioglu, P. J. Valdes, Multiple causes of the Younger dryas cold period. *Nat. Geosci.* **8**, 946–950 (2015).
5. L. D. Trusel, S. B. Das, M. B. Osman, M. J. Evans, B. E. Smith, X. Fettweis, J. R. McConnell, B. P. Y. Noël, M. R. van den Broeke, Nonlinear rise in Greenland runoff in response to post-industrial Arctic warming, *Nature* **564**, 104–108 (2018).
6. O. Heiri, S. J. Brooks, H. Renssen, A. Bedford, M. Hazekamp, B. Ilyashuk, E. S. Jeffers, B. Lang, E. Kirilova, S. Kuiper, L. Millet, S. Samartin, M. Toth, F. Verbruggen, J. E. Watson, N. van Asch, E. Lammertsma, L. Amon, H. H. Birks, H. J. B. Birks, M. F. Mortensen, W. Z. Hoek, E. Magyari, C. M. Sobrino, H. Seppä, W. Tinner, S. Tonkov, S. Veski, A. F. Lotter, Validation of climate model-inferred regional temperature change for late glacial Europe. *Nat. Commun.* **5**, 4914 (2014).
7. F. Schenk, M. Väliranta, F. Muschitiello, L. Tarasov, M. Heikkilä, S. Björck, J. Brandefelt, A. V. Johansson, J.-O. Näslund, B. Wohlfarth, Warm summers during the Younger Dryas cold reversal. *Nat. Commun.* **9**, 1634 (2018).
8. H. Renssen, R. F. B. Isarin, The two major warming phases of the last deglaciation at 14.7 and 11.5 ka cal BP in Europe: Climate reconstruction and AGCM experiments. *Glob. Planet. Ch.* **30**, 117–153 (2001).
9. R. B. Alley, The Younger Dryas cold interval as viewed from central Greenland. *Quat. Sci. Rev.* **19**, 213–226 (2000).
10. H. M. Benway, J. F. McManus, D. W. Oppo, J. L. Cullen, Hydrographic changes in the eastern subpolar North Atlantic during the last deglaciation. *Quat. Sci. Rev.* **29**, 3336–3345 (2010).

11. A. Brauer, C. Endres, C. Günter, T. Litt, M. Stebich, J. F. W. Negendank, High resolution sediment and vegetation responses to Younger Dryas climate change in varved lake sediments from Meerfeld Maar, Germany. *Quat. Sci. Rev.* **18**, 321–329 (1999).
12. J. Bakke, Ø. Lie, E. Heegaard, T. Dokken, G. H. Haug, H. H. Birks, P. Dulski , T. Nilsen, Rapid oceanic and atmospheric changes during the Younger Dryas cold period. *Nat. Geosci.* **2**, 202–205 (2009).
13. D. Hofer, C. C. Raible, A. Dehnert, J. Kuhlemann, The impact of different glacial boundary conditions on atmospheric dynamics and precipitation in the North Atlantic region. *Clim. Past.* **8**, 935–949 (2012).
14. N. Merz, C. C. Raible, T. Woollings, North Atlantic eddy-driven jet in interglacial and glacial winter climates. *J. Climate* **28**, 3977–3997 (2015).
15. J. Zeeberg, The European sand belt in eastern Europe - and comparison of Late Glacial dune orientation with GCM simulation results. *Boreas* **27**, 127–139 (1998).
16. A. Ohmura, P. Kasser, M. Funk, Climate at the equilibrium line of glaciers. *J. Glaciol.* **38**, 397–411 (1992).
17. S. Bony, B. Stevens, D. M. W. Frierson, C. Jakob, M. Kageyama, R. Pincus, T. G. Shepherd, S. C. Sherwood, A. P. Siebesma, A. H. Sobel, M. Watanabe, M. J. Webb, Clouds, circulation and climate sensitivity. *Nat. Geosci.* **8**, 261–268 (2015).
18. J. Kuhlemann, E. J. Rohling, I. Krumrei, P. Kubik, S. Ivy-Ochs, M. Kucera, Regional synthesis of Mediterranean atmospheric circulation during the Last Glacial Maximum. *Science* **321**, 1338–1340 (2008).
19. N. Koç, E. Jansen, H. Haflidason, Paleoceanographic reconstructions of surface ocean conditions in the Greenland, Iceland and Norwegian seas, through the last 14-ka based on diatoms. *Quat. Sci. Rev.* **12**, 115–140 (1993).
20. A. L. C. Hughes, R. Gyllencreutz, Ø. S. Lohne, J. Mangerud, J. I. Svendsen, The last Eurasian ice sheets—A chronological database and time-slice reconstruction, DATED-1. *Boreas* **45**, 1–45 (2016).
21. P. Ludwig, E. J. Schaffernicht, Y. Shao, J. G. Pinto, Regional atmospheric circulation over Europe during the Last Glacial Maximum and its links to precipitation. *J. Geophys. Res. Atmos.* **121**, 2130–2145 (2016).
22. H. Renssen, R. F. B. Isarin, Surface temperature in NW Europe during the Younger Dryas: AGCM simulation compared with temperature reconstructions. *Climate Dynam.* **14**, 33–44 (1998).

23. A. G. Barnston, R. E. Livezey, Classification, seasonality and persistence of low-frequency atmospheric circulation patterns. *Mon. Wea. Rev.* **115**, 1083–1126 (1987).
24. K. M. Nissen, G. C. Leckebusch, J. G. Pinto, D. Renggli, S. Ulbrich, U. Ulbrich, Cyclones causing wind storms in the Mediterranean: Characteristics, trends and links to large-scale patterns. *Nat. Haz. Earth Syst. Sci.* **10**, 1379–1391 (2010).
25. R. Hall, R. Erdélyi, E. Hanna, J. M. Jone, A. A. Scaife, Drivers of North Atlantic Polar Front jet stream variability. *Int. J. Clim.* **35**, 1697–1720 (2015).
26. M. Luetscher, R. Boch, H. Sodemann, C. Spötl, H. Cheng, R. L. Edwards, S. Frisia, F. Hof, W. Müller, North Atlantic storm track changes during the Last Glacial Maximum recorded by Alpine speleothems. *Nat. Comms.* **6**, 6344 (2015).
27. I. S. Castañeda, E. Schefuß, J. Pätzold, J. S. S. Damsté, S. Weldeab, S. Schouten, Millennial-scale sea surface temperature changes in the eastern Mediterranean (Nile River Delta region) over the last 27,000 years. *Paleoceanography* **25**, PA1208 (2010).
28. I. Cacho, J. O. Grimalt, C. Pelejero, M. Canals, F. J. Sierro, J. A. Flores, N. Shackleton, Dansgaard-Oeschger and Heinrich event imprints in Alboran Sea paleotemperatures. *Paleoceanography* **14**, 698–705 (1999).
29. A. Hayes, M. Kucera, N. Kallel, L. Sbaffi, E. J. Rohling, Glacial Mediterranean sea surface temperatures based on planktonic foraminiferal assemblages. *Quat. Sci. Rev.* **24**, 999–1016 (2005).
30. F. Justino, W. R. Peltier, The glacial North Atlantic Oscillation. *Geophys. Res. Lett.* **32**, L21803 (2005).
31. M. R. Haylock, N. Hofstra, A. M. G. Klein Tank, E. J. Klok, P. D. Jones, M. New, A European daily high-resolution gridded data set of surface temperature and precipitation for 1950–2006. *J. Geophys. Res.* **113**, D20119 (2008).
32. D. Rind, D. Peteet, W. Broecker, A. McIntyre, W. Ruddiman, The impact of cold North Atlantic sea surface temperatures on climate: Implications for the Younger Dryas cooling (11–10 k). *Climate Dynam.* **1**, 3–33 (1986).
33. F. He, Simulating transient climate evolution of the last deglaciation with CCSM3, thesis, University of Wisconsin-Madison, USA (2011); http://www.cgd.ucar.edu/ccr/TraCE/doc/He_PhD_dissertation_UW_2011.pdf.
34. L. Menviel, A. Timmermann, O. E. Timm, A. Mouchet, Deconstructing the Last Glacial Maximum termination: The role of millennial and orbital-scale forcings. *Quat. Sci. Rev.* **30**, 1155–1172 (2011).

35. E. Armstrong, P. O. Hopcroft, P. J. Valdes, A simulated Northern Hemisphere terrestrial climate dataset for the past 60,000 years. *Sci. Data* **6**, 265 (2019).
36. M. Porter, Linking of the surface North Atlantic Ocean to adjacent terrestrial ice masses, thesis, University of Aberdeen, UK (2013); http://digitool.abdn.ac.uk:80/webclient/DeliveryManager?application=DIGITOOL-3&owner=resourcediscovery&custom_att_2=simple_viewer&pid=195922.
37. P. D. Hughes, J. C. Woodward, P. C. van Calsteren, L. E. Thomas, K. R. Adamson, Pleistocene ice caps on the coastal mountains of the Adriatic Sea. *Quat. Sci. Rev.* **29**, 3690–3708 (2011).
38. L. M. Baldidni, J. U. L. Baldini, F. McDermott, P. Arias, M. Cueto, I. J. Fairchild, D. L. Hoffmann, D. P. Mattey, W. Müller, D. Constantin Nita, R. Ontañón, C. Garciá-Moncó, D. A. Richards, North Iberian temperature and rainfall seasonality over the Younger Dryas and Holocene. *Quat. Sci. Rev.* **226**, 105998 (2019).
39. A. Moreno, H. Hasenauer, Spatial downscaling of European climate data. *Int. J. Clim.* **36**, 1444–1458 (2016).
40. B. Sevruk, Regional dependency of precipitation-altitude relationship in the Swiss Alps. *Clim. Chge.* **36**, 355–369 (1997).
41. M. Ogrin, E. Kozamernik, Vertical precipitation gradients: A case study of Alpine valleys of northwestern Slovenia. *Theo. App. Clim.* **140**, 401–409 (2020).
42. B. R. Rea, D. J. A. Evans, Quantifying climate and glacier mass balance in North Norway during the Younger Dryas. *Palaeogeogr. Palaeoclimatol. Palaeoecol.* **246**, 307–330 (2007).
43. D. I. Benn, N. R. J. Hulton, An Excel™ spreadsheet program for reconstructing the surface profile of former mountain glaciers and ice caps. *Comput. Geosci.* **36**, 605–610 (2010).
44. C. J. Van der Veen, *Fundamentals of Glacier Dynamics*, CRC Press, Taylor and Francis Group (ed. 2, 2017), 408 p.
45. R. Pellitero, B. R. Rea, M. Spagnolo, J. Bakke, S. Ivy-Ochs, C. R. Frew, P. Hughes, A. Ribolini, S. Lukas, H. Renssen, GlaRe, a GIS tool to reconstruct the 3D surface of palaeoglaciers. *Comp. Geosci.* **94**, 77–85 (2016).
46. J. F. Nye, The mechanics of glacier flow. *J. Glaciol.* **2**, 82–93 (1952).
47. R. Pellitero, B. R. Rea, M. Spagnolo, J. Bakke, P. Hughes, S. Ivy-Ochs, S. Lukas, A. Ribolini, A GIS tool for automatic calculation of glacier equilibrium-line altitudes. *Comput. Geosci.* **82**, 55–62 (2015).

48. B. R. Rea, Defining modern day Area-Altitude-Balance-Ratios (AABRs) and their use in glacier-climate reconstructions. *Quat. Sci. Rev.* **28**, 237–248 (2009).
49. R. F. B. Isarin, Permafrost distribution and temperature in Europe during the Younger Dryas. *Pfrost. Peri. Proc.* **8**, 313–333 (1997).
50. S. A. Elias, Beetle Records: Overview, in *Encyclopaedia of Quaternary Science* (Elsevier Science, 2007), pp. 153–163.
51. H. J. B. Birks, H. Seppä, Pollen-based reconstructions of late-quaternary climate in Europe progress, problems, and pitfalls. *Acta Palaeobotan.* **44**, 317–334 (2004).
52. N. Koç, A. I. Miettinen, C. Stickley, Diatom Records: North Atlantic and Arctic, in *Encyclopaedia of Quaternary Science*, S.A. Elias, Ed. (Elsevier, 2013), pp. 567–576.
53. M. Kucera, M. Weinelt, T. Kiefer, U. Pflaumann, A. Hayes, M. Weinelt, M.-T. Chen, A. C. Mix, T. T. Bárrows, E. Cortijo, J. Duprat, S. Juggins, C. Waelbroeck, Reconstruction of sea-surface temperatures from assemblages of planktonic foraminifera: Multi-technique approach based on geographically constrained calibration data sets and its application to glacial Atlantic and Pacific Oceans. *Quat. Sci. Rev.* **24**, 951–998 (2005).
54. P. Anand, H. Elderfield, M. H. Conte, Calibration of Mg/Ca thermometry in planktonic foraminifera from a sediment trap time series. *Paleoceanography* **18**, 1050 (2003).
55. P. J. Müller, G. Kirst, G. Ruhland, I. von Storch, A. Rosell-Melé, Calibration of the alkenone paleotemperature index $U_{37}^{K'}$ based on core-tops from the eastern South Atlantic and the global ocean (60°N-60°S). *Geochim. Cosmochim. Acta* **62**, 1757–1772 (1998).
56. J.-H. Kim, J. van der Meer, S. Schouten, P. Helmke, V. Willmott, F. Sangiorgi, N. Koç, E. C. Hopmans, J. S. S. Damsté, New indices and calibrations derived from the distribution of crenarchaeal isoprenoid tetraether lipids: Implications for past sea surface temperature reconstructions. *Geochim. Cosmochim. Acta*, **74**, 4639–4654 (2010).
57. R. J. Hijmans, S. E. Cameron, J. L. Parra, P. G. Jones, A. Jarvis, Very high resolution interpolated climate surfaces for global land areas. *Int. J. Clim.* **25**, 1965–1978 (2005).
58. V. Ducić, J. Luković, D. Burić, G. Stanojević, S. Mustafić, Precipitation extremes in the wettest Mediterranean region (Krivošije) and associated atmospheric circulation types. *Nat. Haz. Earth Syst. Sci.* **12**, 687–697 (2012).
59. E. Anderson, S. Harrison, D. G. Passmore, T. M. Mighall, Geomorphic evidence of Younger Dryas glaciation in the Macgillycuddy's Reeks, south west Ireland. *Quat. Proc.* **6**, 75–90 (1998).

60. J. Bakke, S. O. Dahl, Ø. Paasche, R. Løvlie, A. Nesje, Glacier fluctuations, equilibrium-line altitudes and palaeoclimate in Lyngen, northern Norway, during the Lateglacial and Holocene. *The Holocene* **15**, 518–540 (2005).
61. C. K. Ballantyne, A. M. Hall, W. Phillips, S. Binnie, P. W. Kubik, Age and significance of former low-altitude corrie glaciers on Hoy, Orkney Islands, Scot. *J. Geol.* **43**, 107–114 (2007).
62. J. M. Bendle, N. F. Glasser, Palaeoclimatic reconstruction from Lateglacial (Younger Dryas Chronozone) cirque glaciers in Snowdonia, North Wales.*Proc. Geol. Assoc.* **123**, 130–145 (2012).
63. R. Böhlert, M. Egli, M. Maisch, D. Brandova, S. Ivy-Ochs, P. W. Kubik, W. Haeberli, Application of a combination of dating techniques to reconstruct the Lateglacial and early Holocene landscape history of the Albula region (eastern Switzerland). *Geomorphology* **127**, 1–13 (2011).
64. D. Q. Bowen, F. M. Phillips, A. M. McCabe, P. C. Knutz, G. A. Sykes, New data for the Last Glacial Maximum in Great Britain and Ireland. *Quat. Sci. Rev.* **21**, 89–101 (2002).
65. V. H. Brown, D. J. A. Evans, I. S. Evans, The glacial geomorphology and surficial geology of the south-west English Lake District. *J. Maps* **7**, 221–243 (2011).
66. V. H. Brown, D. J. A. Evans, A. Vieli, I. S. Evans, The Younger Dryas in the English Lake District: Reconciling geomorphological evidence with numerical model outputs. *Boreas* **42**, 1022–1042 (2013).
67. R. M. Carrasco, J. Pedraza, D. Domínguez-Villar, J. K. Willenbring, J. Villa, Sequence and chronology of the Cuerpo de Hombre paleoglacier (Iberian Central System) during the last glacial cycle. *Quat. Sci. Rev.* **129**, 163–177 (2015).
68. A. Çiner, M. A. Sarikaya, Cosmogenic ^{36}Cl geochronology of late Quaternary glaciers in the Bolkar Mountains, south central Turkey. *Geol. Soc. Lond. Spec. Pub.* **433**, 271–287 (2017).
69. E. A. Colhoun, F. M. Singe, The Cirque Moraines at Lough Nahanagan, County Wicklow, Ireland. *Proc. R. Ir. Acad.* **80B**, 25–45 (1980).
70. E. Cossart, M. Fort, D. Bourlès, R. Braucher, R. Perrier, L. Siame, Deglaciation pattern during the Lateglacial/Holocene transition in the southern French Alps. Chronological data and geographical reconstruction from the Clarée Valley (upper Durance catchment, southeastern France). *Palaeogeogr. Palaeoclimatol. Palaeoecol.* **315-316**, 109–123 (2012).
71. R. Darnault, Y. Rolland, R. Braucher, D. Bourlès, M. Revel, G. Sanchez, S. Bouissou, Timing of the last deglaciation revealed by receding glaciers at the Alpine-scale: Impact on mountain geomorphology. *Quat. Sci. Rev.* **31**, 127–142 (2012).

72. M. Delmas, Y. Gunnell, R. Braucher, M. Calvet, D. Bourlès, Exposure age chronology of the last glaciation in the eastern Pyrenees. *Quatern. Res.* **69**, 231–241 (2008).
73. J. Dzierżek, Paleogeografia wybranych obszarów Polski w czasie ostatniego zlodowacenia [Paleogeography of Selected Areas of Poland During the Last Glaciation]. *Acta Geograph. Lodzi.* **95**, 1–112 (2009).
74. Z. Engel, P. Mentlík, R. Braucher, J. Minár, L. Léanni, M. Arnold, Geomorphological evidence and ^{10}Be exposure ages for the Last Glacial Maximum and deglaciation of the Velká and Malá Studená dolina valleys in the High Tatra Mountains, central Europe. *Quat. Sci. Rev.* **124**, 106–123 (2015).
75. Z. Engel, R. Braucher, A. Traczyk, L. Laetitia; AsterTeam, ^{10}Be exposure age chronology of the last glaciation in the Krkonoše Mountains, Central Europe. *Geomorph.* **206**, 107–121 (2014).
76. P. R. Federici, D. E. Granger, M. Pappalardo, A. Ribolini, M. Spagnolo, A. J. Cyr, Exposure age dating and Equilibrium Line Altitude reconstruction of an Egesen moraine in the Maritime Alps, Italy. *Boreas* **37**, 245–253 (2008).
77. A. Finlayson, N. Golledge, T. Bradwell, D. Fabel, Evolution of a Lateglacial mountain icecap in northern Scotland. *Boreas* **40**, 536–554 (2011).
78. D. M. Gheorghiu, D. Fabel, J. D. Hansom, S. Xu, Lateglacial surface exposure dating in the Monadhliath Mountains, Central Highlands, Scotland. *Quat. Sci. Rev.* **41**, 132–146 (2012).
79. D. M. Gheorghiu, M. Hosu, C. Corpade, S. Xu, Deglaciation constraints in the Parâng Mountains, Southern Romania, using surface exposure dating. *Quat. Int.* **388**, 156–167 (2015).
80. C. Giraudi, M. Frezzotti, Late pleistocene glacial events in the central Apennines, Italy. *Quat. Res.* **48**, 280–290 (1997).
81. A. Gómez-Ortiz, D. Palacios, B. Palade, L. Vázquez-Selem, F. Salvador-Franch, The deglaciation of the Sierra Nevada (Southern Spain). *Geomorphology* **159-160**, 93–105 (2012).
82. S. Harrison, N. Glasser, E. Anderson, S. Ivy-Ochs, P. W. Kubik, Late Pleistocene mountain glacier response to North Atlantic climate change in southwest Ireland. *Quat. Sci. Rev.* **29**, 3948–3955 (2010).
83. A. Hormes, S. Ivy-Ochs, P. W. Kubik, L. Ferreli, A. M. Michetti, ^{10}Be exposure ages of a rock avalanche and a late glacial moraine in Alta Valtellina, Italian Alps. *Quat. Int.* **190**, 136–145 (2008).
84. P. D. Hughes, R. J. Braithwaite, C. R. Fenton, C. Schnabel, Two Younger Dryas glacier phases in the English Lake District: Geomorphological evidence and preliminary ^{10}Be exposure ages. *North West Geog.* **12**, 10–19 (2012).

P. D. Hughes, C. R. Fenton, P. L. Gibbard, Quaternary Glaciations of the Atlas Mountains, North Africa, *Dev. Quat. Res.* **15**, 1065–1074 (2011).

86. P. D. Hughes, J. C. Woodward, P. C. van Calsteren, L. E. Thomas, The glacial history of the Dinaric Alps, Montenegro. *Quat. Sci. Rev.* **30**, 3393–3412 (2011).
87. J. Ince, Two postglacial pollen profiles from the uplands of Snowdonia, Gwynedd, North Wales. *New Phytol.* **95**, 159–172 (1983).
88. S. Ivy-Ochs, H. Kerschner, A. Reuther, M. Maisch, R. Sailer, J. Schaefer, P. W. Kubik, H.-A. Synal, C. Schlüchter, The timing of glacier advances in the northern European Alps based on surface exposure dating with cosmogenic ^{10}Be, ^{26}Al, ^{36}Cl, and ^{21}Ne. *Geol. Soc. Am. Spec. Paper* **415**, 43–60 (2006).
89. S. Ivy-Ochs, C. Schlüchter, P. W. Kubik, G. H. Denton, Moraine exposure dates imply synchronous Younger Dryas glacier advances in the European Alps and in the Southern Alps of New Zealand. *Geog. Ann.* **81**, 313–323 (1999).
90. M. Jambrina-Enríquez, M. Rico, A. Moreno, M. Leira, P. Bernárdez, R. Prego, C. Recio, B. L. Valero-Garcés, Timing of deglaciation and postglacial environmental dynamics in NW Iberia: The Sanabria Lake record. *Quat. Sci. Rev.* **94**, 136–158 (2104).
91. R. A. Kenyon, The glaciation of the Nephin Beg Range, Co. Mayo, Erie. *Quat. Stud.* **2**, 14–21 (1986).
92. M. Kirkbride, J. Everest, D. Benn, D. Gheorghiu, A. Dawson, Late-Holocene and Younger Dryas glaciers in the northern Cairngorm Mountains, Scotland. *The Holocene* **24**, 141–148 (2014).
93. J. Kuhlemann, M. Milivojević, I. Krumrei, P. W. Kubik, Last glaciation of the Šara range (Balkan Peninsula): Increasing dryness from the LGM to the Holocene. *Aust. J. Earth Sci.* **102**, 146–158 (2009).
94. E. Larsen, M. K. Stalsberg, Younger Dryas glaciolacustrine rhythmites and cirque glacier variations at Kråkenes, western Norway: Depositional processes and climate. *J. Paleolim.* **31**, 49–61 (2004).
95. S. Lukas, T. Bradwell, Reconstruction of a Lateglacial (Younger Dryas) mountain ice field in Sutherland, northwestern Scotland, and its palaeoclimatic implications. *J. Quat. Sci.* **25**, 567–580 (2010).
96. M. Makos, J. Nitychoruk, M. Zreda, The Younger Dryas climatic conditions in the Za Mnichem Valley (Polish High Tatra Mountains) based on exposure-age dating and glacier-climate modelling. *Boreas* **42**, 745–761 (2013).

97. A. P. Moran, S. Ivy-Ochs, M. Schuh, M. Christl, H. Kerschner, Evidence of central Alpine glacier advances during the Younger Dryas– early Holocene transition period. *Boreas* **45**, 398–410 (2016).
98. Ø. Paasche, S. Olaf Dahl, J. Bakke, R. Løvlie, A. Nesje, Cirque glacier activity in arctic Norway during the last deglaciation. *Quatern. Res.* **68**, 387–399 (2007).
99. D. Palacios, N. de Andrés, J. de Marcos, L. Vázquez-Selem, Glacial landforms and their paleoclimatic significance in Sierra de Guadarrama, Central Iberian Peninsula. *Geomorph.* **139-140**, 67–78 (2012).
100. D. Palacios, N. de Andrés, J. I. López-Moreno, J. M. García-Ruiz, Late Pleistocene deglaciation in the upper Gállego Valley, central Pyrenees. *Quatern. Res.* **83**, 397–414 (2015).
101. R. Pallas, A. Rodés, R. Braucher, J. Carcaillet, M. Ortuño, J. Bordonau, D. Bourlès, J. M. Vilaplana, E. Masana, P. Santanach, Late Pleistocene and Holocene glaciation in the Pyrenees: A critical review and new evidence from ^{10}Be exposure ages, south-central Pyrenees. *Quat. Sci. Rev.* **25**, 2937–2963 (2006).
102. R. Pallas, Á. Rodés, R. Braucher, D. Bourlès, M. Delmas, M. Calvet, Y. Gunnell, Small, isolated glacial catchments as priority targets for cosmogenic surface exposure dating of Pleistocene climate fluctuations, southeastern Pyrenees. *Geol.* **38**, 891–894 (2010).
103. D. Pearce, Reconstruction of Younger Dryas glaciation in the Tweedsmuir Hills, Southern Uplands, Scotland: Style, dynamics and palaeo-climatic implications, thesis, University of Worcester, UK (2014).
104. F. M. Phillips, D. Q. Bowen, D. Elmore, Surface exposure dating of glacial features in Great Britain using cosmogenic chlorine-36: Preliminary results. *Min. Mag.* **58A**, 722–723 (1994).
105. R. J. Pope, P. D. Hughes, E. Skourtsos, Glacial history of Mt Chelmos, Peloponnesus, Greece. *Geol. Soc. Lond. Spec. Pub.* **433**, 211–236 (2015).
106. I. Schindelwig, N. Akçar, P. W. Kubik, C. Schlüchter, Lateglacial and early Holocene dynamics of adjacent valley glaciers in the Western Swiss Alps. *J. Quat. Sci.* **27**, 114–124 (2012).
107. E. Serrano, J. J. González-Trueba, M. González-García, Mountain glaciation and paleoclimate reconstruction in the Picos de Europa (Iberian Peninsula, SW Europe). *Quatern. Res.* **78**, 313–314 (2012).
108. D. Small, V. Rinterknecht, W. Austin, D. Fabel, M. Miguens-Rodriguez, S. Xu, *In situ* cosmogenic exposure ages from the Isle of Skye, northwest Scotland: Implications for the timing of deglaciation and readvance from 15 to 11 ka. *J. Quat. Sci.* **27**, 150–158 (2012).

109. M. R. Standell, Lateglacial (Younger Dryas) Glaciers and Ice-Sheet Deglaciation in the Cairngorm Mountains, Scotland: Glacier Reconstructions and their Palaeoclimatic Implications, thesis, University of Loughborough, UK (2014).

110. M. J. C. Walker, Early-and mid-flandrian environmental history of the Brecon Beacons, South Wales. *N. Phyto.* **91**, 147–165 (1982).

111. R. Walker, Diatom and pollen studies of a sediment profile from Melynllyn, a Mountain Tarn in Snowdonia, North Wales. *N. Phyto.* **81**, 791–804 (1978).

112. K. R. Wilson, The last glaciation in the Western Mourne Mountains, Northern Ireland. *Scot. Geog. J.* **120**, 199–210 (2004).

113. P. Wilson, Evidence for and Reconstruction of a Nahanagan Stade Glacier at Croloughan Lough, Derryveagh Mountains, Co. Donegal. *Ir. J. Earth. Sci.* **22**, 45–54 (2004).

114. C. Zahno, N. Akçar, V. Yavuz, P. W. Kubik, C. Schlüchter, Chronology of Late Pleistocene glacier variations at the Uludağ Mountain, NW Turkey. *Quat. Sci. Rev.* **29**, 1173–1187 (2010).

115. M. Zreda, A. Çiner, M. A. Sarikaya, C. Zweck, S. Bayari, Remarkably extensive glaciation and fast deglaciation and climate change in Turkey near the Pleistocene-Holocene boundary. *Geol.* **39**, 1051–1054 (2011).

116. M. V. A. Martins, A. R. Perretti, E. Salgueiro, F. Frontalini, J. Moreno, A. M. Soares, M. Mahiques, S. Silva, C. A. de Azevedo, J. A. Dias, Atlantic sea surface temperatures estimated from planktonic foraminifera off the Iberian Margin over the last 40Ka BP. *Mar. Geol.* **367**, 191–201 (2015).

117. L. Amon, S. Veski, A. Heinsalu, L. Saarse, Timing of Lateglacial vegetation dynamics and respective palaeoenvironmental conditions in southern Estonia: Evidence from the sediment record of Lake Nakri. *J. Quat. Sci.* **27**, 169–180 (2012).

118. A. C. Ashworth, The climatic significance of a late Quaternary insect fauna from Rodbaston Hall, Staffordshire, England. *Insect Syst. Evol.* **4**, 191–205 (1973).

119. C. J. A. Birks, N. Koç, A high-resolution diatom record of late-Quaternary sea-surface temperatures and oceanographic conditions from the eastern Norwegian Sea. *Boreas* **31**, 323–344 (2002).

120. H. H. Birks, V. J. Jones, S. J. Brooks, H. J. B. Birks, R. J. Telford, S. Juggins, S. M. Peglar, From cold to cool in northernmost Norway: Lateglacial and early Holocene multi-proxy environmental and climate constructions from Jansvatnet, Hammerfest.*Quat. Sci. Rev.* **33**, 100–120 (2012).

121. W. W. Bishop, G. R. Coope, Stratigraphical and faunal evidence for lateglacial and early flandrian environments in south-west Scotland, in *Studies in the Scottish Lateglacial Environment*, J. M. Gray, J. J. Lowe, Eds. (Pergamon, 1977), pp. 61–88.

122. S. Bohncke, J. Vandenberghe, A. S. Huijzer, Periglacial environments during the Weichselian Late Glacial in the Maas Valley, the Netherlands. *Geol. Mijn.* **72**, 193–210 (1993).

123. A. Bordon, O. Peyron, A.-M. Lézine, S. Brewer, E. Fouache, Pollen-inferred Late-Glacial and Holocene climate in southern Balkans (Lake Maliq), *Quat. Int.* **200**, 19–30 (2009).

124. S. J. Brooks, I. P. Matthews, H. H. Birks, H. J. B. Birks, High resolution Lateglacial and early-Holocene summer air temperature records from Scotland inferred from chironomid assemblages. *Quat. Sci. Rev.* **41**, 67–82 (2012).

125. P. Cabedo-Sanz, S. T. Belt, J. Knies, K. Husum, Identification of contrasting seasonal sea ice conditions during the Younger Dryas. *Quat. Sci. Rev.* **79**, 74–86 (2013).

126. I. Cacho, J. O. Grimalt, M. Canals, L. Sbaffi, N. J. Shackleton, J. Schönfeld, R. Zahn, Variability of the western Mediterranean Sea surface temperature during the last 25,000 years and its connection with the Northern Hemisphere climatic changes. *Paleoceanography* **16**, 40–52 (2001).

127. N. Combourieu-Nebout, O. Peyron, V. Bout-Roumazeilles, S. Goring, I. Dormoy, S. Joannin, L. Sadori, G. Siani, M. Magny, Holocene vegetation and climate changes in the central Mediterranean inferred from a high-resolution marine pollen record (Adriatic Sea). *Clim. Past* **9**, 2023–2042 (2013).

128. G. R. Coope, G. Lemdahl, J. J. Lowe, A. Walkling, Temperature gradients in northern Europe during the last glacial–Holocene transition (14-9 ^{14}C kyr BP) interpreted from coleopteran assemblages. *J. Quat. Sci.* **13**, 419–433 (1998).

129. G. R. Coope, M. J. Joachim, Lateglacial environmental changes interpreted from fossil Coleoptera from St Bees, Cumbria, England, in *Studies in the Lateglacial of North-West Europe*, J. J. Lowe, J. M. Gray, J. E. Robinson, Eds. (Pergamon, 1980), pp. 55–68.

130. G. R. Coope, J. H. Dickson, J. A. McCutcheon, G. F. Mitchell, The Lateglacial and Early Postglacial Deposit at Drumurcher, Co. Monaghan *Proc. Roy. Ir. Acad.* **79**, 63–85 (1979).

131. P. Coxon, P. O'Callaghan, The distribution and age of pingo remnants in Ireland, in *Periglacial Processes and Landforms in Britain and Ireland*, J. Boardman, Ed. (Cambridge Univ. Press, 1987), pp. 195–201.

132. I. Dormoy, O. Peyron, N. Combourieu Nebout, S. Goring, U. Kotthoff, M. Magny, J. Pross, Terrestrial climate variability and seasonality changes in the Mediterranean region between 15000 and 4000 years BP deduced from marine pollen records. *Clim. Past* **5**, 615–632 (2009).
133. T. D. Douglas, Periglacial involutions and the evidence for coversands in the English Midlands. *Proc. Yor. Geol. Soc.* **44**, 131–143 (1982).
134. H. Ebbesen, M. Hald, Unstable Younger Dryas climate in the northeast North Atlantic. *Geol. 32, 673–676 (2004).*
135. T. Eldevik, B. Risebrobakken, A. E. Bjune, C. Andersson, H. J. B. Birks, T. M. Dokken, H. Drange, M. S. Glessmer, C. Li, J. E. Ø. Nilsen, O. H. Otterå, K. Richter, Ø. Skagseth, A brief history of climate—The northern seas from the Last Glacial Maximum to global warming. *Quat. Sci. Rev.* **106**, 225–246 (2014).
136. L. Essallami, M. A. Sicre, N. Kallel, L. Labeyrie, G. Siani, Hydrological changes in the Mediterranean Sea over the last 30,000 years. *Geochem. Geoph. Geosys.* **8**, Q07002 (2007).
137. J. Frechen, E. A. Rosauer, Aufbau und Gliederung des Wiirm-Loess-Profils van Kärlich in Neuwieder Becken. *Fortschr. Geol. Rheinl. Westf.* **4**, 267–282 (1959).
A. Gogou, I. Bouloubassi, V. Lykousis, M. Arnaboldi, P. Gaitani, P. A. Meyers, Organic geochemical evidence of Late Glacial–Holocene climate instability in the North Aegean Sea. *Palaeogeogr. Palaeoclimatol. Palaeoecol.* **256**, 1–20 (2007).
139. O. Heiri, M.-L. Filippi, A. F. Lotter, Lateglacial summer temperature in the Trentino area (Northern Italy). *Studi Trent. Sci. Nat. Acta Geol.* **82**, 299–308 (2005).
140. O. Heiri, H. Cremer, S. Engels, W. Z. Hoek, W. Peeters, A. F. Lotter, Lateglacial summer temperatures in the Northwest European lowlands: A chironomid record from Hijkermeer, the Netherlands. *Quat. Sci. Rev.* **26**, 2420–2437 (2007).
141. O. Heiri, L. Millet, Reconstruction of Late Glacial summer temperatures from chironomid assemblages in Lac Lautrey (Jura, France). *J. Quat. Sci.* **20**, 33–44 (2005).
142. O. Heiri, W. Tinner, A. F. Lotter, Evidence for cooler European summers during periods of changing meltwater flux to the North Atlantic. *Proc. Nat. Acad. Sci.* **101**, 15285–15288 (2004).
143. I. Heyse, Cryoturbation types in Eolian Würm late glacial sediments in Flanders, Belgium. *Polarforschung* **53**, 87–95 (1983).

144. B. Huntley, W. A. Watts, J. R. M. Allen, B. Zolitschka, Palaeoclimate, chronology and vegetation history of the Weichselian Lateglacial: Comparative analysis of data from three cores at Lago Grande di Monticchio, southern Italy. *Quat. Sci. Rev.* **18**, 945–960 (1999).
145. E. S. Jeffers, M. B. Bonsall, S. J. Brooks, K. J. Willis, Abrupt environmental changes drive shifts in tree–grass interaction outcomes. *J. Ecol.* **99**, 1063–1070 (2011).
146. M. J. Joachim, Late-glacial coleopteran assemblages from the west coast of the Isle of Man, thesis, University of Birmingham, UK (1978).
147. N. Koç Karpuz, E. Jansen, A high-resolution diatom record of the last deglaciation from the SE Norwegian Sea: Documentation of rapid climatic changes. *Paleoceanography* **7**, 499–520 (1992).
148. E. Kolstrup, A fossil frost mound of Late Dryas age in middle Jutland (Denmark). *Boreas* **14**, 217–223 (1985).
149. U. Kotthoff, A. Koutsodendris, J. Pross, G. Schmiedl, A. Bornemann, C. Kaul, G. Marino, O. Peyron, R. Schiebel, Impact of Lateglacial cold events on the northern Aegean region reconstructed from marine and terrestrial proxy data. *J. Quat. Sci.* **26**, 86–96 (2011).
150. B. Lang, S. J. Brooks, A. Bedford, R. T. Jones, H. J. B. Birks, J. D. Marshall, Regional consistency in Lateglacial chironomid-inferred temperatures from five sites in north-west England. *Quat. Sci. Rev.* **29**, 1528–1538 (2010).
151. I. Larocque, W. Finsinger, Late-glacial chironomid-based temperature reconstructions for Lago Piccolo di Avigliana in the southwestern Alps (Italy). *Palaeogeogr. Palaeoclimatol. Palaeoecol.* **257**, 207–223 (2008).
152. G. Lemdahl, Late-glacial and early-Holocene Coleoptera assemblages as indicators of local environment and climate at Kråkenes Lake, western Norway. *J. Paleolim.* **23**, 57–66 (2000).
153. G. Lemdahl, A rapid climatic change at the end of the Younger Dryas in south Sweden—Palaeoclimatic and palaeoenvironmental reconstructions based on fossil insect assemblages. *Palaeogeogr. Palaeoclimatol. Palaeoecol.* **83**, 313–331 (1991).
154. B. Lyashuk, E. Gobet, O. Heiri, A. F. Lotter, J. F. N. van Leeuwen, W. O. van der Knaap, E. Ilyashuk, F. Oberli, B. Ammann, Lateglacial environmental and climatic changes at the Maloja Pass, Central Swiss Alps, as recorded by chironomids and pollen. *Quat. Sci. Rev.* **28**, 1340–1353 (2009).
155. M. Magny, J. Guiot, P. Schoellammer, Quantitative reconstruction of Younger Dryas to mid-holocene paleoclimates at Le Locle, Swiss Jura, using pollen and lake-level data. *Quat. Res.* **56**, 170–180 (2001).

156. B. Martrat, J. O. Grimalt, N. J. Shackleton, L. de Abreu, M. A. Hutterli, T. F. Stocker, Four climate cycles of recurring deep and surface water destabilizations on the Iberian Margin. *Science* **317**, 502–507 (2007).
157. B. Martrat, P. Jimenez-Amat, R. Zahn, J. O. Grimalt, Similarities and dissimilarities between the last two deglaciations and interglaciations in the North Atlantic region. *Quat. Sci. Rev.* **99**, 122–134 (2014).
158. G. Ménot, E. Bard, A precise search for drastic temperature shifts of the past 40,000 years in southeastern Europe. *Paleoceanography* **27**, PA2210 (2012).
L. Millet, D. Rius, D. Galop, O. Heiri, S. J. Brooks, Chironomid-based reconstruction of Lateglacial summer temperatures from the Ech palaeolake record (French western Pyrenees). *Palaeogeogr. Palaeoclimatol. Palaeoecol.* **315**, 86–99 (2012).
160. C. Muñoz Sobrino, O. Heiri, M. Hazekamp, D. van der Velden, E. P. Kirilova, I. García-Moreiras, A. F. Lotter, New data on the Lateglacial period of SW Europe: A high resolution multiproxy record from Laguna de la Roya (NW Iberia). *Quat. Sci. Rev.* **80**, 58–77 (2013).
161. A. Nesje, J. Bakke, S. J. Brooks, D. S. Kaufman, E. Kihlberg, M. Trachsel, W. J. D'Andrea, J. A. Matthews, Late glacial and Holocene environmental changes inferred from sediments in Lake Myklevatnet, Nordfjord, western Norway. *Veg. Hist. Archbot.* **23**, 229–248 (2014).
162. M. Cristina Peñalba, M. Arnold, J. Guiot, J.-C. Duplessy, J.-L. Beaulieu, Termination of the last glaciation in the Iberian Peninsula inferred from the pollen sequence of Quintanar de la Sierra. *Quatern. Res.* **48**, 205–214 (1997).
163. A. Penaud, F. Eynaud, J.L. Turon, D. Blamart, L. Rossignol, F. Marret, C. Lopez-Martinez, J.O. Grimalt, B. Malaizé, K. Charlier, Contrasting paleoceanographic conditions off Morocco during Heinrich events (1 and 2) and the Last Glacial Maximum. *Quat. Sci. Rev.* **29**, 1923–1939 (2010).
164. O. Peyron, C. Bégeot, S. Brewer, O. Heiri, M. Magny, L. Millet, P. Ruffaldi, E. Van Campo, G. Yu, Late-Glacial climatic changes in Eastern France (Lake Lautrey) from pollen, lake-levels, and chironomids. *Quatern. Res.* **64**, 197–211 (2005).
165. A. Pissart, Remnants of periglacial mounds in the Hautes Fagnes (Belgium): Structure and age of the ramparts. *Geol. Mijn.* **62**, 551–555 (1983).
166. M. Rodrigo-Gámiz, F. Martínez-Ruiz, S. W. Rampen, S. Schouten, J. S. Sinninghe Damsté, Sea surface temperature variations in the western Mediterranean Sea over the last 20 kyr: A dual-organic proxy ($U^{K'}_{37}$ and LDI) approach. *Paleoceanography* **29**, 87–98 (2014).

167. E. Salgueiro, F. Naughton, A. H. L. Voelker, L. de Abreu, A. Alberto, L. Rossignol, J. Duprat, V. H. Magalhães, S. Vaqueiro, J.-L. Turon, F. Abrantes, Past circulation along the western Iberian margin: A time slice vision from the Last Glacial to the Holocene. *Quat. Sci. Rev.* **106**, 316–329 (2014).
168. S. Samartin, O. Heiri, A. F. Lotter, W. Tinner, Climate warming and vegetation response after Heinrich event 1 (16 700–16 000 cal yr BP) in Europe south of the Alps. *Clim. Past* **8**, 1913–1927 (2012).
169. S. Samartin, O. Heiri, E. Vescovi, S. J. Brooks, W. Tinner, Lateglacial and early Holocene summer temperatures in the southern Swiss Alps reconstructed using fossil chironomids. *J. Quat. Sci.* **27**, 279–289 (2012).
170. F. W. Shotton, G. R. Coope, Exposures in the power house terrace of the river stour at Wilden, Worcestershire, England.*Proc. Geol. Ass.* **94**, 33–44 (1983).
171. B. W. Sparks, R. B. G. Williams, F. G. Bell, Presumed ground-ice depressions in East Anglia. *Proc. R. Soc. Lond. A* **327**, 329–343 (1972).
172. H. Svensson, Relict periglacial structures. Occurrences, age and development in different matrices on a coastal plain of Southwestern Sweden. *Geog. Ann. A* **72**, 79–91 (1990).
173. M. Tóth, E. K. Magyari, S. J. Brooks, M. Braun, K. Buczkó, M. Bálint, O. Heiri, A chironomid-based reconstruction of late glacial summer temperatures in the southern Carpathians (Romania). *Quatern. Res.* **77**, 122–131 (2012).
174. N. Van Asch, M. E. Kloos, O. Heiri, P. de Klerk, W. Z. Hoek, The younger dryas cooling in northeast Germany: Summer temperature and environmental changes in the Friedländer Groβe Wiese region. *J. Quat. Sci.* **27**, 531–543 (2012).
175. N. van Asch, A. F. Lutz, M. C. H. Duijkers, O. Heiri, S. J. Brooks, W. Z. Hoek, Rapid climate change during the Weichselian Lateglacial in Ireland: Chironomid-inferred summer temperatures from Fiddaun, Co. Galway. *Palaeogeogr. Palaeoclimatol. Palaeoecol.* **315-316**, 1–11 (2012).
176. B. Van Geel, G. R. Coope, T. Van Der Hammen, Palaeocology and stratigraphy of the Lateglacial type section at Usselo (The Netherlands). *Rev. Pal. Pal.* **60**, 25–129 (1989).
177. M. J. Walker, G. R. Coope, C. Sheldrick, C. S. Turney, J. J. Lowe, S. P. E.. Blockley, D. D. Harkness, Devensian Lateglacial environmental changes in Britain: A multi-proxy environmental record from Llanilid, South Wales, UK. *Quat. Sci. Rev.* **22**, 475–520 (2003).

178. M. J. C. Walker, G. R. Coope, J. J. Lowe, The Devensian (Weichselian) Lateglacial palaeoenvironmental record from Gransmoor, East Yorkshire, England: A contribution to the 'North Atlantic seaboard programme' of IGCP-253, 'Termination of the Pleistocene'. *Quat. Sci. Rev.* **12**, 659–680 (1993).
179. E. Watson, Remains of pingos in wales and the Isle of man. *Geol. J.* **7**, 381–392 (1971).
180. J. E. Watson, S. J. Brooks, N. J. Whitehouse, P. J. Reimer, H. J. B. Birks, C. Turney, Chironomid-inferred late-glacial summer air temperatures from Lough Nadourcan, Co. Donegal, Ireland. *J. Quat. Sci.* **25**, 1200–1210 (2010).
